# Topological Lasing from Thouless Pumping in Bilayer Photonic Crystal

**DOI:** 10.1021/acsphotonics.5c02664

**Published:** 2026-02-11

**Authors:** D.-H.-Minh Nguyen, Dung Xuan Nguyen, H. Chau Nguyen, Thibaud Louvet, Emmanuel Drouard, Xavier Letartre, Dario Bercioux, Hai Son Nguyen

**Affiliations:** † 226245 Donostia International Physics Center, Donostia-San Sebastián 20018, Spain; ‡ Advanced Polymers and Materials: Physics, Chemistry and Technology, Chemistry Faculty (UPV/EHU), Paseo M. Lardizabal 3, San Sebastian 20018, Spain; § Brown Theoretical Physics Center and Department of Physics, Brown University, 182 Hope Street, Providence, Rhode Island 02912, United States; ∥ Center for Theoretical Physics of Complex Systems, 364806 Institute for Basic Science (IBS), Daejeon 34126, Republic of Korea; ⊥ Naturwissenschaftlich-Technische Fakultät, Universität Siegen, Walter-Flex-Straße 3, Siegen 57068, Germany; # Ecole Centrale de Lyon, INSA Lyon, 52864 Université Claude Bernard Lyon 1, CPE Lyon, CNRS, INL, UMR5270, Ecully 69130, France; ¶ IKERBASQUE, Basque Foundation for Science, Euskadi Plaza, 5, Bilbao 48009, Spain; ∇ IUF, Universié de France, Paris 75231, France

**Keywords:** topological lasing, photonic grating, reconfigurable lasing, phase change material, topological phase transition

## Abstract

Topological lasing leverages concepts from topological physics to achieve single-mode light amplification within topological bandgaps, offering robustness against fabrication imperfections. Recent advances in microelectromechanical systems (MEMSs) and phase-change materials (PCMs) at the subwavelength scale promise new avenues for dynamically reconfigurable topological lasers, enabling robust and tunable nanoscale light sources. Here, we numerically demonstrate a dynamically reconfigurable lasing action at telecom wavelengths in a bilayer photonic crystal through the mechanisms of Thouless pumping. By designing two competing periodic potentials—one slowly translating photonic grating atop another stationary one—we observe a transition between a topological pumping regime and conventional mode oscillation. A carefully engineered heterojunction between these phases supports a robust lasing mode that can be dynamically tuned via MEMSs or reversible PCMs. Our work establishes bilayer photonic crystals as a programmable platform for achieving topological light sources, showcasing a potential pathway for merging topological photonics with reconfigurable photonic devices.

## Graphical abstract

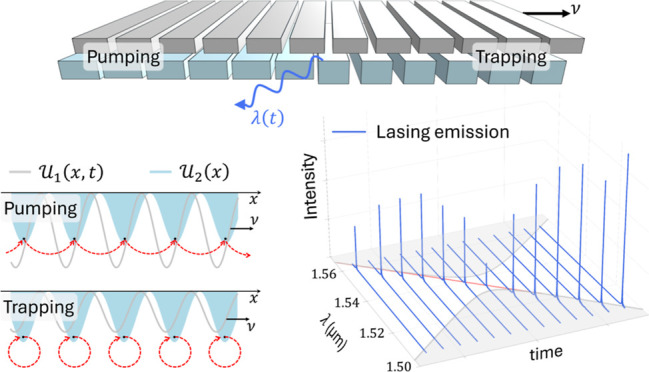

## Introduction

Topological photonics offers powerful tools to engineer novel states of light, using topological protection to create robust optoelectronic devices.^1–6^ A prominent example is topological lasing, where light amplification occurs in edge states immune to disorder, offering enhanced robustness against fabrication imperfections. These systems typically rely on edge modes, such as those from topological insulators,^7–9^ Jackiw–Rebbi interface states,^10–15^ or valley–Hall effects.^16–22^ However, most demonstrations to date have focused on static platforms with fixed geometries, lacking reconfigurability—a key limitation for applications requiring adaptive or programmable functionalities.^23–25^ Meanwhile, another cornerstone of topological physics is the model of Thouless pumping, which presents quantized transport driven by a slowly varying potential,^26^ has never been considered in this perspective. Although Thouless pumping has been demonstrated in photonic systems^27–32^ —most notably in coupled waveguide arrays—its integration into practical devices like lasers or LEDs remains elusive.

Recent advances in bilayer photonic crystal slabs have opened new opportunities for light control, enabling phenomena such as moiré photonics,^33,34^ chiral responses,^35,36^ optical singularities,^37,38^ asymmetric radiating metasurfaces,^39–41^ and synthetic momenta for topological physics.^42,43^ Crucially, the ability to dynamically shift one layer relative to the other using microelectromechanical systems (MEMS) offers a unique mechanism to tailor band structures and realize tunable photonic functionalities.^44,45^ An alternative approach for achieving reconfigurability is through the integration of phase-change materials (PCMs),^25^ such as germanium-antimony-tellurium (GST) or antimony trisulfide (Sb_2_S_3_). These materials exhibit reversible transitions between amorphous and crystalline phases, accompanied by pronounced changes in their optical properties. Incorporating PCMs into photonic structures enables dynamic control of light–matter interactions, making possible nonvolatile switching, reconfigurable metasurfaces, and even programmable topological phases.

Here, inspired by a Thouless pumping model in a bipartite potential, we demonstrate the tunability of a topological lasing mode by designing a heterojunction configuration incorporating PCMs. This heterojunction supports a high-Q topological interface mode, which can be dynamically tuned using MEMSs or switched by inducing the crystal-to-amorphous transition in the PCM. Our numerical findings extend Thouless’s fundamental concept into a versatile photonic platform, providing a blueprint for engineering photonic devices that exploit topological robustness and tunability for advanced light manipulation and lasing applications.

## Results and Discussion

### Conceptual Overview

Thouless pumping can be illustrated by considering spinless particles in a one-dimensional (1D) space subject to a time- and space-dependent periodic potential

U(x,t)=U(x+Λ,t)=U(x,t+T)

where Λ and *T* are the periodicity in space and time, respectively. There are *N*_*p*_ particles in each minimum of the potential. For simplicity, we assume *N*_*p*_ = 1 and that each particle is in its ground state inside a well. As this potential is adiabatically shifted along the *x*-axis, the wells carry the particles with them, resulting in a displacement Λ of the particles per time period *T*. Consequently, the integral of the current over a time period is an integer value, which is 1, giving rise to quantized transport. The adiabatic condition is met if the potential changes slowly enough that the particles remain in their ground state throughout the process.

In his seminal work,^26^ Thouless proposed an example of U(x,t) as a superposition of two periodic potentials: $U\left(x,t\right)=U_{1}\left(x,t\right)+U_{2}\left(x\right)$ . Here, $U_{1}\left(x,t\right)=U_{1}\left(x-\nu t\right)$ is a potential sliding slowly at a velocity ν whereas $U_{2}\left(x\right)$ remains stationary. Both potentials share the same spatial period Λ, as depicted in Figure 1a,b. Qian Niu later showed that the electrons in filled bands are locked into the stronger component of the bipartite potential.^46^ Specifically, the particles’ motion depends on the competition between the mobile $U_{1}\left(x,t\right)$ and the stationary $U_{2}\left(x\right)$ . While $U_{1}\left(x,t\right)$ tends to push the particles to induce pumping, $U_{2}\left(x\right)$ exerts a counteracting force and tends to localize them. When the driving potential $U_{1}\left(x,t\right)$ dominates, the particles are transported by a distance Λ at the end of a pumping cycle, as depicted in Figure 1a,c. Conversely, if the stationary potential is stronger (Figure 1b), the particles return to their original positions at *t* = *T* = Λ/ν—see Figure 1d. We term the latter regime “trapping” of particles. Readers may refer to the Supplemental Video 1 for a dynamic visualization of Figure 1a,b.

**Figure 1:**
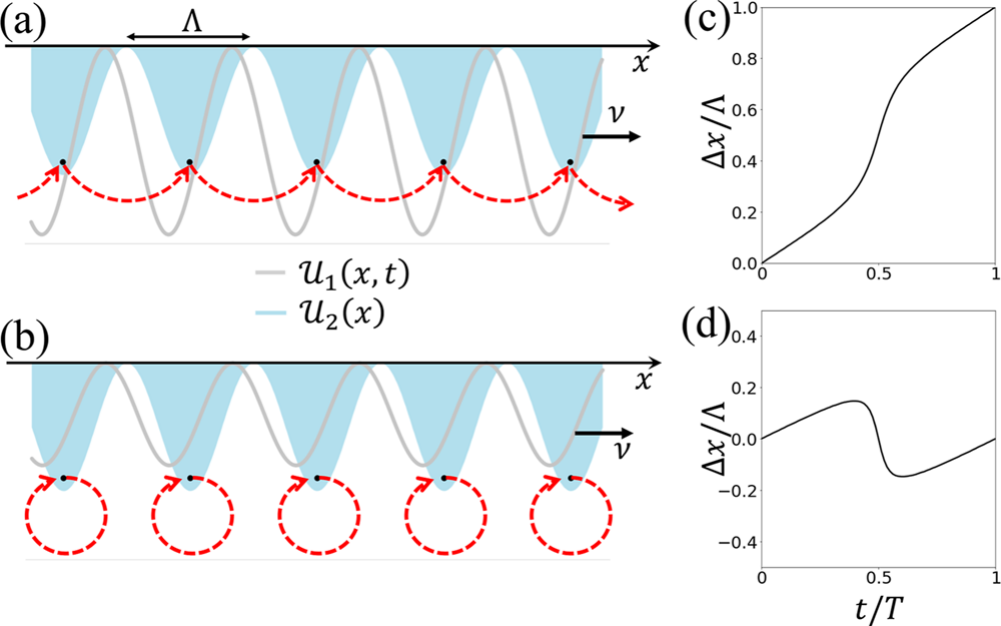
Pumping versus trapping. (a,b) Schematic drawings of two periodic potentials of the same periodicity where $U_{1}\left(x,t\right)$ moves slowly and $U_{2}\left(x\right)$ is stationary. The particles (denoted by black dots) can either be transported by $U_{1}\left(x,t\right)$ to the next unit cell (a), or be pulled back by $U_{2}\left(x\right)$ to their original positions (b). (c,d) Exemplary changes in position of a particle in each case during a pumping cycle with sine potentials $U_{1}\left(x,t\right)=2.2\;\sin\left(2\pi x/\Lambda -2\pi \nu t/\Lambda \right)$ (c) [ $U_{1}\left(x,t\right)=1.2\;\sin\left(2\pi x/\Lambda -2\pi \nu t/\Lambda \right)$ (d)] and $U_{2}\left(x\right)=1.5\;\sin\left(2\pi x/\Lambda \right)$ .

### Bilayer Photonic Crystal

We propose a realization of the bipartite potential described above in a 1D bilayer photonic crystal that comprises two parallel, high-contrast gratings separated by a distance *d*, as depicted in Figure 2a. The two gratings share the same period Λ and are laterally displaced by δ. They have thicknesses $h_{l}$ , widths $w_{l}$ , and refractive indices $n_{l}$ , where l = 1, 2. All geometrical parameters are of subwavelength scale. As we shall see later, the upper and lower layers represent the potentials $U_{1}\left(x,t\right)$ and $U_{2}\left(x\right)$ , respectively.

**Figure 2:**
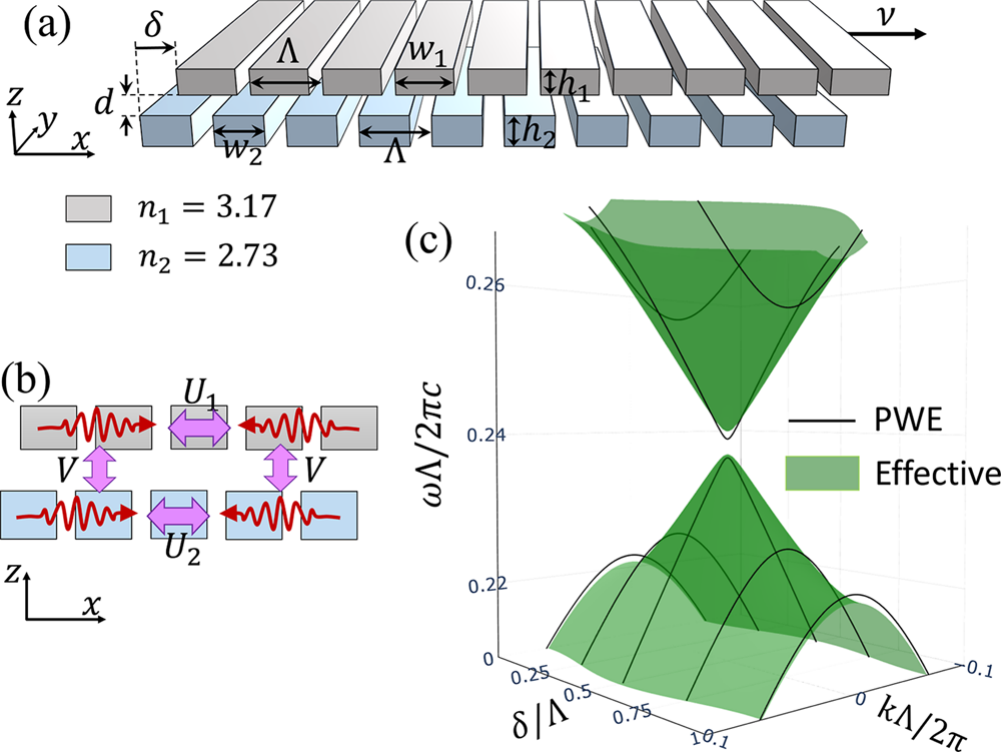
Bilayer photonic crystal. (a) A bilayer photonic crystal composed of two gratings corrugated along the *x* direction with the same period Λ. The gratings are made of materials with refractive indices *n*_1_ and *n*_2_. The upper layer slides slowly at velocity ν, resulting in a lateral displacement δ = νt relative to the lower layer at time *t*. (b) Sketch of guided plane waves (red arrows) and their intra- and interlayer coupling mechanisms. (c) Dispersion of the two lowest guided TE bands of config. (1) for various values of δ. The green surfaces represent the effective model, while the black lines correspond to PWE simulations. Parameters: *w*_1_ = 0.8Λ, *h*_1_ = 0.3Λ, *w*_2_ = 0.81Λ, *h*_2_ = 0.46Λ, and *d* = 0.1Λ.

We focus on the transverse electric (TE) modes, whose *E*_*y*_-components can be described by four guided plane waves: two traveling right and two traveling left, within the upper and lower layers—see Figure 2b. In each layer, diffraction causes counter-propagating modes traveling with velocities ± $v_{l}$ to couple at wave vector *k*_*x*_ = π/Λ (i.e., the *X* point of the first Brillouin zone) with a strength $U_{l}$ that depends on the grating’s geometry and material. Plane waves traveling in the same direction but in different layers interact through their evanescent fields with strength *V*. This coupling amplitude can be adjusted by varying the interlayer separation *d*. The effective Hamiltonian describing the four lowest-frequency guided modes reads^47–49^

1 $$H\left(k,\delta \right)=\left(\begin{array}{llll} \omega_{1}+v_{1}k & U_{1}e^{-i2\pi \delta /\Lambda } & V & 0\\ U_{1}e^{i2\pi \delta /\Lambda } & \omega_{1}-v_{1}k & 0 & V\\ V & 0 & \omega_{2}+v_{2}k & U_{2}\\ 0 & V & U_{2} & \omega_{2}-v_{2}k \end{array}\right)$$

where *k* = *k*_*x*_ + π/Λ is the wave vector measured from the *X* point; ω_1_ and ω_2_ are the frequency offsets in the upper and lower gratings. The phase *e* ^±i2πδ/Λ^ in the intralayer coupling of the upper layer is induced by its displacement δ—see the Supporting Information (SI) for a detailed derivation of the effective Hamiltonian.

We are interested in the two lowest TE-guided modes. Computing the Chern number of the lower gap separating these two modes in some typical cases, we find that the two cases *U*_1_ > *U*_2_ and *U*_1_ < *U*_2_ correspond to the pumping and trapping regimes in the bilayer photonic crystal, respectively. Although numerous designs of the bilayer crystal would yield similar results, we choose two exemplary configurations: config. (1) with (*w*_1_, *h*_1_, *w*_2_, *h*_2_) = (0.8, 0.3, 0.81, 0.46)­Λ, and config. (2) with (*w*_1_, *h*_1_, *w*_2_, *h*_2_) = (0.8, 0.3, 0.77, 0.5)­Λ. The former configuration has *U*_1_ > *U*_2_, and the latter has *U*_1_ < *U*_2_. The interlayer distance is fixed at *d* = 0.1Λ. In both cases, the upper and lower gratings are made of materials with refractive indices *n*_1_ = 3.17 and *n*_2_ = 2.73, respectively. Potential materials for *n*_1_ are amorphous silicon (a-Si), indium phosphide (InP), and gallium arsenide (GaAs), while the refractive index *n*_2_ can be realized in titanium dioxide (TiO_2_), amorphous antimony trisulfide (Sb_2_S_3_) or aluminum arsenide (AlAs).

For the bilayer photonic crystal to experience all possible displacement configurations, we slowly translate the upper layer along the *x*-axis at a velocity ν ≪ $v_{l}$ so that δ = νt (Figure 2a). The adiabatic condition is ensured since we choose the velocity to be sufficiently small so that the photonic modes at each instant are still accurately described by Hamiltonian (1), with negligible influence from the motion. Owing to the lattice translation symmetry, the system is invariant under the transformation δ → δ + Λ. The black lines in Figure 2c show the two lowest bands of config. (1) for several values of δ, simulated using the plane wave expansion (PWE) method with the MIT Photonic Bands package.^50^ By fitting the effective model’s dispersion to the PWE simulation results at δ = 0, we retrieve all necessary parameters (see SI for details), and then plot the two lowest bands of Hamiltonian (1) in Figure 2c (green surfaces), which shows good agreement between the effective model and PWE simulations. The two bands remain separated for all values of δ. The lowest band reaches a maximum while the other minimizes at *k* = 0 and δ = 0.5Λ.

### Photonic Pumping and Trapping

We now show that the bilayer photonic crystal realizes the pumping and trapping regimes, with the mobile upper layer representing $U_{1}\left(x,t\right)$ and the stationary lower layer corresponding to $U_{2}\left(x\right)$ . However, since we cannot define any “particle” in such a photonic crystal, what is pumped or trapped here is the electromagnetic field localized in the dielectric rods of this crystal, that is, energy. To track the motion of this localized field within a unit cell during a pumping cycle, we choose its center to be the Wannier center.^51,52^ The change in position of the field’s center is given by

2 $$\Delta x_{W}\left(t\right)=-\frac{\Lambda }{2\pi }\int_{0}^{t}dt^{\prime }\int_{-\pi /\Lambda }^{+\pi /\Lambda }dk\Omega \left(k,t^{\prime }\right)$$

where $\Omega \left(k,t\right)=i\left(\left\langle \frac{\partial u_{1}}{\partial k}|\frac{\partial u_{1}}{\partial t}\right\rangle -\left\langle \frac{\partial u_{1}}{\partial t}|\frac{\partial u_{1}}{\partial k}\right\rangle \right)$ is the Berry curvature, and |*u*_1_⟩ represents the eigenstate of the lowest-frequency band. At the end of the pumping cycle, *x*_*W*_ changes by Λ if the localized field is successfully pumped, and remains unchanged (i.e., Δ*x*_*W*_ = 0) if it is trapped.

The change in position of the Wannier center for both configurations (1) and (2) is efficiently computed using the effective model and visualized in Figure 3. Indeed, we see that the Wannier center shifts to the next unit cell in config. (1), signifying pumping (Figure 3a), while it returns to its initial position at *t* = *T* in config. (2), indicating trapping [Figure 3b]. We look at the electric field distribution of the lowest mode obtained from PWE simulations to gain a deeper insight into how the fields vary. In both configurations, the electric field localizes in the dielectric rods of both layers across a wide range of wavenumber *k*, with the strongest localization at *k* = 0—see the insets of Figure 3a,b. However, different behaviors emerge: In config. (1), the field follows the upper layer and gets dragged to the next unit cell, analogous to a particle pulled by the potential $U_{1}\left(x,t\right)$ . In config. (2), the localized field tends to stay in the initial unit cell with the lower layer, despite being constantly driven by the upper layer. The electric field’s behavior resembles that of a particle perturbed by $U_{1}\left(x,t\right)$ but constantly pulled back by $U_{2}\left(x\right)$ . Therefore, depending on the configuration, the bilayer photonic crystal can emulate either Thouless pumping or trapping of electromagnetic field—see the Supplemental Video 2 for animations of the insets in Figure 3.

**Figure 3:**
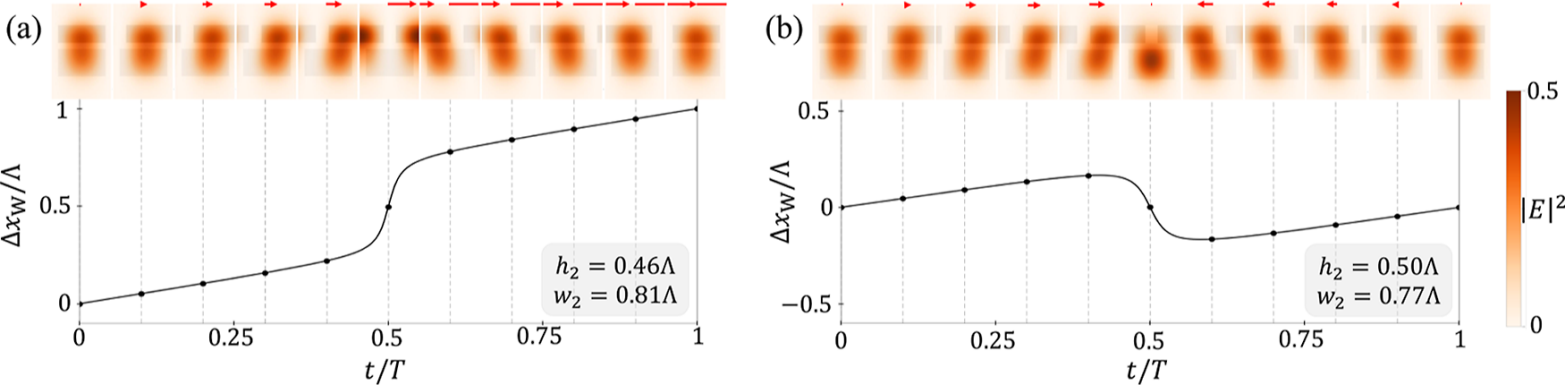
Photonic pumping and trapping. (a,b) Pumping and trapping of localized electric field in bilayer photonic crystal. The position of the localized field is denoted by the variation of the Wannier center, Δ*x*_*W*_, within a unit cell during a pumping period *T* = Λ/ν. If Δ*x*_*W*_ changes by Λ (a) or remains unchanged (b) at the end of the period, the field’s center moves to the next unit cell or returns to its original position, respectively. The upper insets show the electric field distribution within a unit cell of the lowest mode at *k* = 0 at various moments, obtained from PWE simulations. The red arrows indicate the corresponding displacement of the field. Here, the upper layer of the photonic crystal has *h*_1_ = 0.3Λ and *w*_1_ = 0.8Λ in both cases (a and b), and the parameters of the lower layer are shown in the gray insets. The color bar indicates the modulus squared of the normalized electric field.^53^

### Reconfigurable Interface Mode

In the context of Thouless pumping, by considering time (*t*) as an additional dimension, the system can be examined in a (1 + 1)-D parameter space. Within this framework, the energy bands of the lattice are characterized by an invariant known as the Chern number,^28,51^ *C*_*n*_ = −Δ*x*_*Wn*_(*T*)/Λ with *n* the band index, that identifies the topological phase of the system. In our case, the pumping and trapping regimes have different Chern numbers for the lowest band: *C* = −1 and *C* = 0, respectively, indicating two distinct topological phases in the (1 + 1)-D space. By constructing a heterojunction of two photonic crystals with different topological phases, we expect to observe a robust interface mode protected by the topological phase transition across this heterojunction. This interface mode is pumped through the frequency gap as the lattice varies in time.^51^ Hence, we consider a photonic heterojunction composed of config. (1) on the left (L) and config. (2) on the right (R), as illustrated in Figure 4a. Since the upper layer of this heterojunction is a homogeneous grating, it can slide adiabatically at a velocity ν without breaking the invariance of the system under the transformation δ → δ + Λ.

**Figure 4:**
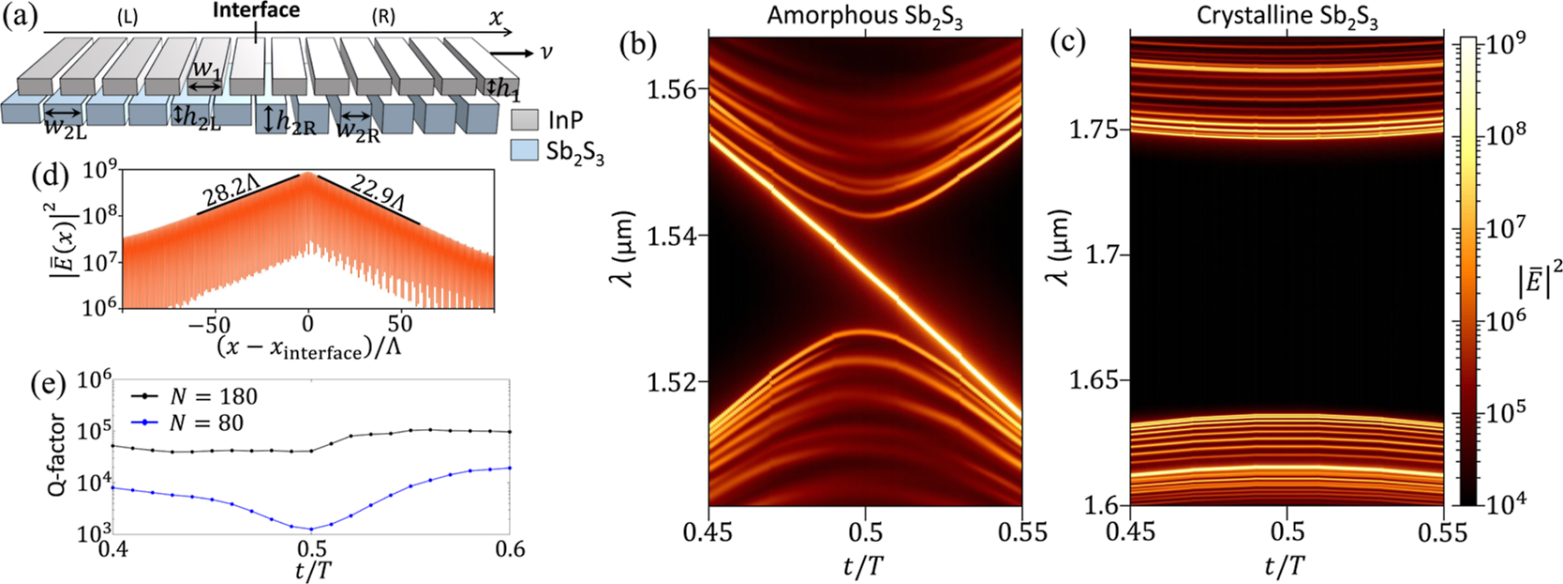
Heterojunction of bilayer photonic crystal. (a) A photonic heterojunction in which the homogeneous upper grating slides with velocity ν while the heterogeneous Sb_2_S_3_ grating is stationary with heights and widths different on either side. The upper layer has *w*_1_ = 0.8Λ and *h*_1_ = 0.3Λ. The left (right) side of the Sb_2_S_3_ layer has *w*_2L(R)_ = 0.81(0.77)­Λ and *h*_2L(R)_ = 0.46(0.5)­Λ. The interlayer distance is *d* = 0.1Λ. (b,c) The spectra of TE modes in the heterojunction when Sb_2_S_3_ is in amorphous (b) and crystalline (c) phases, simulated by FDTD method with the number of periods on each side being *N* = 200. (d) The electric field profile ${\left|\mathrm{E̅}\left(x\right)\right|}^{2}$ of the interface mode in (b) at *t* = 0.5*T*, integrated over the *z* direction. (e) The quality factor of the interface mode in (b) with respect to time for two heterojunction sizes: *N* = 180 and *N* = 80. The lattice period is Λ = 366 nm.

The spectrum of this photonic heterojunction is obtained using the Finite-Difference Time-Domain (FDTD) method implemented in the solver 3D Electromagnetic Simulator of the commercial software Lumerical—see Figure 4b. As the upper layer slides to the right, a distinct mode, absent in the spectra of either config. (1) or (2) individually, traverses the spectral gap from one band to the other. In the vicinity of *t* = 0.5*T*, it sharply contrasts with other modes for having a consistent descent in wavelength. Conversely, if the upper layer slides to the left, this mode’s wavelength monotonically increases. This phenomenon is known for the soliton mode in the Rice–Mele model^28,51^ and can be interpreted as a chiral edge mode along the synthetic dimension.^43^ Indeed, by plotting the field distribution of this mode in Figure 4d, we see that it strongly localizes at the interface of the heterojunction and exponentially decays into the constituent crystals with mismatched decay lengths. The decay lengths differ because the dispersions of the two crystals are different. This interface mode is robust against any perturbations that preserve the bulk spectral gap of both sides, for example, see Figure S10 of the SI.

Since the interface mode acts as a cavity that confines electromagnetic field, we quantify this confinement by the mode’s quality (*Q*) factor. Figure 4e shows the *Q*-factor of the interface mode at various moments for two heterojunctions of different sizes. For the smaller heterojunction, this quantity varies greatly over time, or equivalently, the interlayer displacement δ: It becomes smallest when the interface mode lies at the center of the spectral gap. This dependence wanes as the system size increases, and the *Q*-factor can attain values as high as 10^5^. This suggests potential applications of this photonic heterojunction in devices, such as lasers, beam emitter,^54^ and filters. A demonstration of this photonic heterojunction as a filter is provided in the SI.

### Dynamical Control of Topological Phases

While the wavelength of the topological interface mode is continuously tunable via the adjustment of the lateral displacement δ, this mode can also be switched on and off by inducing a topological transition on one side of the heterojunction. Using the picture of two competing potentials, we see that in the left side of the heterojunction (Figure 4a) the optical mode is pulled by a moving potential of the upper layer. If the potential of the lower layer is sufficiently enhanced to dominate over the upper one, the left side transitions into the trapping regime while the right side’s regime remains unchanged. With both sides of the heterojunction being in the same regime, the interface mode vanishes.

Motivated by a recent observation of topological phase transition in photonic crystal using a PCM,^55^ we demonstrate this idea in our bilayer lattice by incorporating a PCM into its design.^56–59^ Specifically, the lower grating can be fabricated using amorphous antimony trisulfide (Sb_2_S_3_),^60^ an earth-abundant and nontoxic PCM with ultralow losses.^61,62^ Sb_2_S_3_ is stable at room temperature in both its amorphous (*n*_2_ = 2.73) and crystalline (*n*_2_ = 3.26) phases, which can be changed reversibly by either heating the entire sample or selectively illuminating it with laser pulses. The change in refractive index of the lower layer when Sb_2_S_3_ transitions alters the coupling strengths between the guided waves,^63^ enhancing the stationary potential created by the lower layer. Thus, it may induce a topological phase transition in the bilayer photonic crystal, switching it between the pumping and trapping regimes (see the SI for a complete topological phase diagram). This phenomenon indeed occurs in our current photonic heterojunction where, upon the crystallization of Sb_2_S_3_, the photonic crystal on the left side of the heterojunction changes from the pumping to trapping regime while the right side remains in the trapping regime. The spectrum of the lowest spectral gap in the vicinity of *t* = 0.5*T* is shown in Figure 4c when Sb_2_S_3_ crystallizes completely. The gap widens and shifts to longer wavelength compared to Figure 4b since the refractive index of the lower grating increases. Importantly, the interface mode traversing the gap disappears as the two sides of the heterojunction now share the same topology, which serves as a clear signature of the topological phase transition. This also illustrates how PCMs can be used to dynamically switch on and off an optical interface mode.

### Topological Lasing

Owing to the high *Q*-factor and strong confinement of our photonic heterojunction, we can incorporate active (i.e., gain) materials into one layer and perform nonresonant optical excitation to induce lasing at the interface mode.^2,18,64^ The lasing wavelength can be selectively and continuously varied through the dynamical sliding motion of the upper layer. In particular, we use the design of a photonic junction presented before (Figure 4a) with the upper grating composed of InP and InAsP quantum wells (QWs). This layer is continuously illuminated by a nonresonant optical source of wavelength λ_*e*_ = 850 nm (Figure 5a). As sketched in Figure 5b, the pump injects hot carriers to the conduction and valence bands of InP. These hot electrons and holes then relax to the fundamental states of the QWs and then recombine radiatively, leading to a spontaneous emission centered at wavelength λ_*s*_ = 1500 nm. In the spontaneous emission, only photons of wavelength λ_*L*_ associated with the interface mode are in resonance and confined within the cavity; those of other wavelengths decay rapidly. After the electronic population inversion is established, lasing emission can be achieved precisely at the wavelength λ_*L*_.

**Figure 5:**
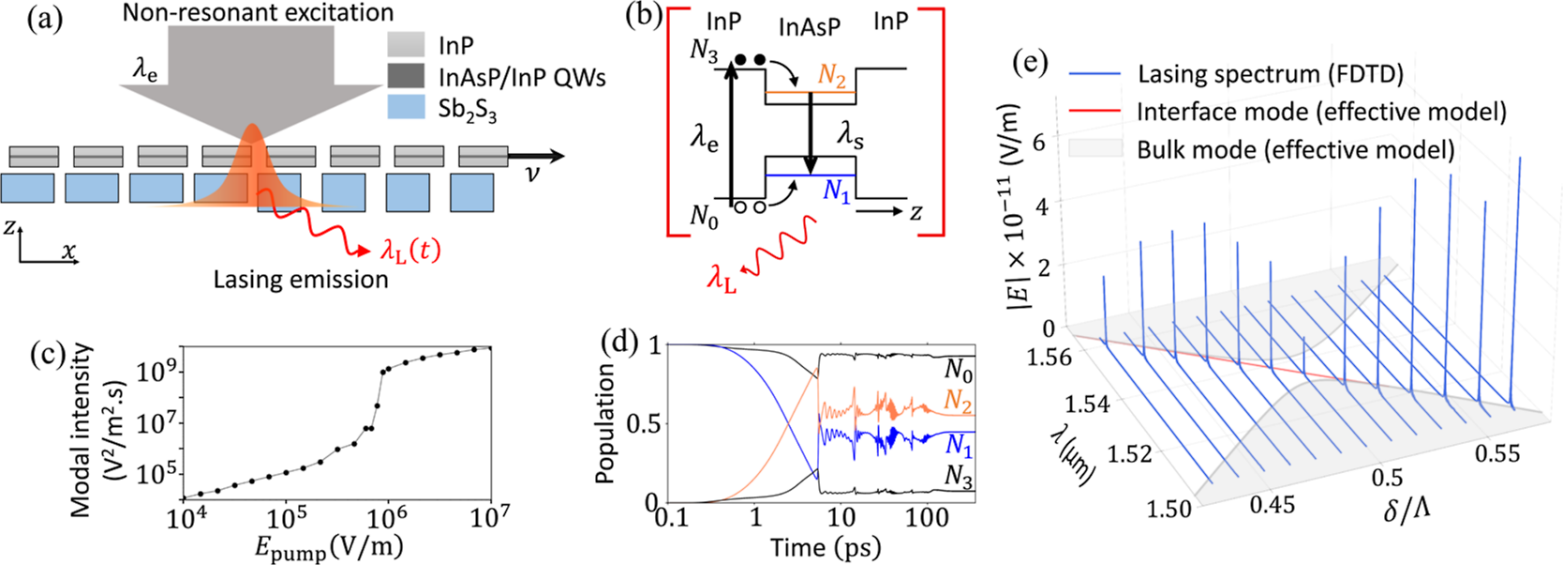
Reconfigurable topological lasing. (a) The heterojunction of bilayer photonic crystal where the upper layer is made of gain material InAsP/InP moves slowly with velocity ν. The heterojunction is continuously pumped by a nonresonant source of wavelength λ_*e*_ = 850 nm and achieves lasing action at λ_*L*_. (b) Schematic diagram of the four-level model for the lasing action with the spontaneous emission wavelength λ_*s*_ = 1500 nm. (c) Dependence of modal intensity of the lasing signal at δ = 0.5Λ on the pump electric field strength. (d) Evolution in time of electron populations at the four levels at *E*_pump_ = 1.3 × 10^6^ V m^–1^. (e) Spectrum of the lasing mode at several moments *t* when the upper layer slides and the nonresonant source has the same strength as (c). The gray line and areas indicate the interface and bulk modes following the effective model. The FDTD simulations are performed with *N* = 150.

We numerically validate this idea through FDTD simulations with the gain material modeled by a four-level two-electron material.^65^ Two levels with electron populations *N*_0_ and *N*_3_ are the band edges of the barrier InP while *N*_1_ and *N*_2_ are two levels of the quantum well InAsP (Figure 5b). We examine the lasing action with the two layers displaced by δ = 0.5Λ. By increasing the field strength of the pump, we observe an emission peak at λ_*L*_ for *E*_pump_ ≳ 7 × 10^5^ V/m. The dependence of the lasing modal intensity on the pump field strength is shown in Figure 5c with the characteristic laser threshold behavior—a clear transition from spontaneous to stimulated emission. Here, the modal intensity is defined by $\frac{1}{2\pi }\int d\omega |E{|}^{2}$ with the integration taken over the frequency range ω encompassing the lasing peak. The temporal evolution of the electron populations at field strength 1.3 × 10^6^ V m^–1^ is shown in Figure 5d, where the population inversion between level 1 and 2 is achieved. The steady state is reached at around 0.16 ns after the excitation and the gain material becomes transparent, giving rise to lasing at the edge mode.

Finally, translating the upper layer slowly at a fixed pump intensity yields the emission spectrum at various moments *t* shown in Figure 5e, where the lasing peaks align with the interface mode. The variation of the lasing wavelength with respect to time is locked to the sliding direction of the upper layer. The spectrum obtained from the effective model is presented as a visual reference showing the interface mode’s variation; it matches perfectly with the FDTD simulation of Figure 4b. A slight discontinuity at δ = 0.5Λ, accompanied by a dip in the emission field strength, is present due to the finite size of the simulated structure (see the SI for more remarks). The presence of spontaneous emission in the spectrum at *t* ≲ 0.5*T* means that the lasing threshold varies with respect to the relative displacement δ. Single-mode lasing across a range of wavelengths can be achieved by constantly keeping the pump power above the threshold. This demonstrates the tunability of the lasing mode in this photonic heterojunction. Such a lasing mode is robust against defects and disorders since they are topologically protected. The single-mode operation is guaranteed as the number of edge mode is one, which is dictated by the change of Chern number across the heterojunction.

## Conclusion

Regarding the experimental feasibility, the bilayer photonic crystal can be fabricated using standard nanofabrication methods, such as electron beam lithography and ionic dry etching.^33,34,41,66^ Dynamic control over the vertical and lateral degrees of freedom can be facilitated by MEMSs.^67–70^ Especially, a recent MEMS integrated into a bilayer photonic lattice has demonstrated its capability to dynamically tune various degrees of freedom, including the interlayer spacing, relative rotation, lateral translation, tilting, and stretching.^45^ The operation speed of MEMS is negligible compared with the speed of light, guaranteeing the adiabatic pumping of the lasing mode. The phase of the PCM Sb_2_S_3_ can be reversibly switched using state of the art microheaters,^71^ such as indium–tin-oxide heater,^72^ silicon PIN diode heater,^73^ or graphene-based heater.^74^

Our proposal of combining MEMS and PCMs for dynamically controlling topological interface modes demonstrates the potential of this bilayer photonic heterojunction for realizing multidimensionally reconfigurable photonic devices. As applications, this includes lasers, beam emitters and filters, providing unprecedented mechanisms of creating and manipulating light. Fundamentally, our results also lay the groundwork for further investigations into 2D Thouless pumping, which is connected to the 4D quantum Hall effect,^75^ and for examining Thouless pumping in moiré lattices, as predicted in twisted bilayer graphene^76,77^ and noted by Thouless himself.^26^ Furthermore, this study opens avenues for exploring novel aspects of Thouless pumping beyond the adiabatic regime^78^ and even in the relativistic regime, where the grating motion approaches the speed of light.^79,80^

## Experimental/Methods

### PWE Simulations

The PWE simulations in this work are carried out by the MIT Photonic Bands package^50^ with a 2D computational cell of size (*L*_*x*_, *L*_*z*_) = (1, 5)­Λ. The resolution is 64 pixels per Λ. The number of bands computed is eight.

### FDTD Simulations

The FDTD simulations in this work are carried out by either the MEEP package^81^ or the commercial software Lumerical.^82^

#### Spectrum

The spectra shown in Figure 4b,c of the photonic heterojunction are obtained from Lumerical FDTD simulations. A dielectric photonic junction is constructed following the geometry depicted in Figure 4a with its interface lying at the center of the computational cell. The refractive indices of the upper and lower gratings are 3.17 and 2.73, respectively. In this linear regime, the parameters scale with the lattice constant Λ, so we set Λ = 1 μm for simplicity. The total number of periods is 400, that is, the length of the heterojunction is 400 μm. The 2D computational cell is enclosed in standard phase-matching layers. The mesh for finite-difference calculation has the maximum mesh step 0.02 μm along the *x* direction and 67 mesh cells per wavelength along the *z* direction. The electromagnetic modes of the system are excited by 20 electric dipoles randomly distributed in the bilayer within a range of 160 μm around the interface. The dipoles are aligned along the *y* axis (θ = 0), have random phases and random angle with respect to the *x* axis. Each of them emits a broadband pulse with frequency ranging from 69 to 74 THz. The simulation runs for 70 ps at 300 K. All signals are recorded and analyzed by 20 time monitors randomly distributed in the system within a range of 240 μm around the interface. We note that the spectra in Figure 4 have a few discrete patterns. They are numerical artifacts stemming from the dielectric gratings crossing a mesh line.

#### Quality Factor

The quality factor of the edge mode is computed using MEEP and Lumerical simulations, with both methods yielding comparable results. In the MEEP simulations, a dielectric photonic junction is constructed similar to that in Lumerical. A single point source, emitting a Gaussian pulse with a frequency width of Δ*f* = 0.002­(*c*/Λ), is randomly embedded in a dielectric rod at the interface. The central frequency of this optical pulse follows a straight trajectory along the chiral edge mode, *f*_center_ = (0.06δ + 0.2086)­(*c*/Λ). The source excites modes with an electric field parallel to the dielectric rod. A monitor is placed inside another dielectric rod at the junction interface to analyze the response for 10^4^ time units after the source has turned off. The 2D computational cell has a resolution of 32 and dimensions of (*N* + 7, 26), where *N* is the number of periods on each side. The boundary layers perpendicular to the *y*-axis are phase matching layers of thickness 2, while those normal to the *x*-axis are adiabatic absorbers of thickness 7. The periodic lattices submerge into the absorbers.

#### Lasing Simulation

For lasing simulations in Lumerical FDTD, the heterojunction is constructed similarly but we use a realistic geometry with Λ = 366 nm since the calculations are nonlinear. The lower grating is a dielectric with the refractive index 2.73 while the upper grating now is modeled by a 4-level 2-electron material,^65^ akin to what is depicted in Figure 5b. In this gain material, the transition wavelengths are λ_*s*_ = 1.5 μm and λ_*e*_ = 0.85 μm, the damping coefficients are γ_*a*_ = γ_*b*_ = 10^13^ Hz, the lifetimes of different decay channels are *t*_30_ = *t*_21_ = 3 × 10^–10^ s and *t*_32_ = *t*_10_ = 10^–13^ s, and the electron population density is 1 × 10^23^ m^–3^. The heterojunction is continuously pumped by a spatial-Gaussian beam with wavelength λ_*e*_ and waist radius 2 μm, located 2.2 μm above the system. The signals are recorded and analyzed by 10 time monitors located 0.5 μm below the system. The heterojunction in these simulations has 300 periods, corresponding to a length of approximately 110 μm. The simulations run for 360 ps at 300 K. The mesh of the finite-difference method has the maximum mesh step 0.006 μm along the *x* direction and 60 mesh cells per wavelength along the *z* direction. More details about the lasing simulations can be found in the SI.

## Supplementary Material







## Notes

Supplemental video 1: Thouless pumping and trapping in a bipartite periodic potential (MP4)
Supplemental video 2: Thouless pumping and trapping in bilayer photonic gratings (MP4)
Supporting Information: Derivation of the effective Hamiltonian, topological phase diagram obtained by incorporating the phase-change material, experimental data and fit function of antimony trisulfide’s temperature dependence, proposal for a reconfigurable broadband filtering, details about the FDTD simulations, captions of the supplemental videos, and supplemental figures (PDF)

## Abbreviations

MEMSs: microelectromechanical systems
PCM: phase-change material
SI: supporting information
TE: transverse electric
PWE: plane wave expansion
FDTD: finite-difference time-domain
QW: quantum well
