# Supplementary material for: Topological Lasing from Thouless Pumping in Bilayer Photonic Crystal
Source: ACS Photonics. 2026 Feb 11;13(5):1399–406. doi: 10.1021/acsphotonics.5c02664 (PMC12964543; doi:10.1021/acsphotonics.5c02664)
Supplement: Supplementary file 3 [file ph5c02664_si_003.pdf]

Topological Lasing from Thouless Pumping in Bilayer Photonic Crystal: Supplemental Information

D.-H.-Minh Nguyen*[1,2], Dung Xuan Nguyen*[3,4], H. Chau Nguyen[5], Thibaud Louvet[6], Emmanuel Drouard[6], Xavier Letartre[6], Dario Bercioux*[1,7], and Hai Son Nguyen*[6,8]

[1]Donostia International Physics Center, Donostia-San Sebastián 20018, Spain
[2]Advanced Polymers and Materials: Physics, Chemistry and Technology, Chemistry Faculty (UPV/EHU), Paseo M. Lardizabal 3, San Sebastian 20018, Spain
[3]Brown Theoretical Physics Center and Department of Physics, Brown University, 182 Hope Street, Providence, Rhode Island 02912, USA
[4]Center for Theoretical Physics of Complex Systems, Institute for Basic Science (IBS), Daejeon 34126, Republic of Korea
[5]Naturwissenschaftlich-Technische Fakultät, Universität Siegen, Walter-Flex-Straße 3, Siegen 57068, Germany
[6]Ecole Centrale de Lyon, INSA Lyon, Université Claude Bernard Lyon 1, CPE Lyon, CNRS, INL, UMR5270, Ecully 69130, France
[7]IKERBASQUE, Basque Foundation for Science, Euskadi Plaza, 5, Bilbao 48009, Spain
[8]IUF, Universié de France, Paris 75231, France

*Email: dnguyen001@ikasle.ehu.eus (DHMN), dungmuop@gmail.com (DXN), dario.bercioux@dipc.org (DB), hai-son.nguyen@ec-lyon.fr (HSN)

Contents

Effective Hamiltonian 2

Dynamic Control of Topological Phases via Phase-Change Material 6

Temperature Dependence of Antimony Trisulfide 7

Guided Transmission for Broadband Filtering 8

Finite-Difference Time-Domain Simulations 9

Edge State from Effective Model 12

Captions of Supplemental Videos 12

Supplemental Figures 12

Effective Hamiltonian

In this section, we present the derivation of the effective Hamiltonian. The system consists of two gratings with the same period Λ separated by distance D. The confinement of electromagnetic waves within each grating is considerably analogous to the problem of an electron in a finite quantum well. Hence, for simplicity, we will investigate how the optical modes in each grating are effectively described and then phenomenologically add the evanescent coupling between modes in different gratings.

We consider a symmetric grating of dielectric constant ε_s in the air with thickness H and width L, the spatial dielectric function is given by $\varepsilon(x,z) = [\varepsilon(x) - 1]f_{\mathrm{e}}(z) + 1$, where the relative dielectric constant of the grating with respect to the environment is a periodic function with the period Λ and

$$\varepsilon(x) = \left[\begin{array}{cc} \varepsilon_s & \text{for } -L/2 < x < L/2 \\ 1 & \text{for } -\Lambda/2 < x < -L/2 \text{ or } L/2 < x < \Lambda/2 \end{array}\right., \tag{S1}$$

and $f_{\mathrm{e}}(z) = \Theta(z+W/2) - \Theta(z-W/2)$ [1]. As $\varepsilon(x) - 1$ is a periodic function, we can write its Fourier expansion as $\varepsilon(x) - 1 = \sum_{n=-\infty}^{+\infty} \xi_n e^{i2\pi nx/\Lambda}$, which gives

$$\varepsilon(x,z) = \xi_0 f_{\mathrm{e}}(z) + 1 + \sum_{n\neq 0} \xi_n(z) e^{i\frac{2\pi n}{\Lambda}x} = \bar{\varepsilon}(z) + \sum_{n\neq 0} \xi_n(z) e^{i\frac{2\pi n}{\Lambda}x}, \qquad \xi_n(z) = \xi_n f_{\mathrm{e}}(z). \tag{S2}$$

The electromagnetic field of this system is governed by the Maxwell's equations

$$\begin{aligned} \nabla \cdot \mathbf{H} = 0, \qquad \nabla \times \mathbf{H} = \varepsilon_0 \varepsilon \frac{\partial \mathbf{E}}{\partial t}, \\ \nabla \cdot (\varepsilon \mathbf{E}) = 0, \qquad \nabla \times \mathbf{E} = -\mu_0 \frac{\partial \mathbf{H}}{\partial t}. \end{aligned}$$

Since the system is uniform and infinite along the y direction, we can decompose the solutions at momentum $k_y = 0$ into two sets of modes: transverse electric (TE) modes with $E_x = E_z = 0$, and transverse magnetic (TM) modes with $H_x = H_z = 0$. These modes have fields' strengths distribute uniformly along the y direction. In our case, we are only interested in TE modes but a theory for the TM ones can be developed similarly. The Maxwell's equations are consequently reduced to the wave equation

$$\frac{\partial^2 E_y}{\partial z^2} + \frac{\partial^2 E_y}{\partial x^2} = -\varepsilon(x,z)\frac{\omega^2}{c^2} E_y. \tag{S3}$$

Due to the discrete translation symmetry of the grating, we employ the Bloch theorem to write the electric field in terms of plane waves

$$E_y(\mathbf{r}) = \sum_n C_n(z) e^{i(k_x + K_n)x}, \qquad \text{with } K_n = \frac{2\pi n}{\Lambda}. \tag{S4}$$

Inserting this expression into the wave equation, we get

$$\sum_n e^{i(k_x+K_n)x} \left[\frac{\partial^2}{\partial z^2} + \bar{\varepsilon}(z)\frac{\omega^2}{c^2} - (k_x + K_n)^2\right] C_n(z) = -\frac{\omega^2}{c^2} \sum_{n,l\neq 0} \xi_l(z) e^{i(k_x+K_{n+l})x} C_n(z). \tag{S5}$$

Multiply two sides with $e^{-i(k_x+K_m)x}$ and integrate over x, we get

$$\left[\frac{\partial^2}{\partial z^2} + \bar{\varepsilon}(z)\frac{\omega^2}{c^2} - (k_x + K_n)^2\right] C_n(z) = -\frac{\omega^2}{c^2} \sum_{l\neq n} \xi_{n-l}(z) C_l(z). \tag{S6}$$

We divide the plane wave basis into two sets: basic waves $\mathcal{B}$ and others $\mathcal{O}$. The basic waves are those that contribute most to the modes of interest. We impose our first approximation by assuming that the basic waves

[1] $\Theta(x)$ is the Heaviside function.

have the form $C_n(z) = E_{0y}(z)C_n$ for $n \in \mathcal{B}$, where $E_{0y}(z)$ is the envelop function of the lowest-frequency guided TE modes in a homogeneous slab described by the dielectric function $\bar{\varepsilon}(z)$. We have

$$\frac{\partial^2 E_{0y}(z)}{\partial z^2} - (k_x + K_n)^2 E_{0y}(z) = -\bar{\varepsilon}(z)\frac{\omega^2}{c^2}E_{0y}(z). \tag{S7}$$

Here, ω and $E_{0y}(z)$ vary with respect to the momentum $k_x + K_n$. Since in the case of grating, we only work with a small range of momentum around a high-symmetry point $k_x + K_n = \tilde{K}$, we assume that ω and $E_{0y}(z)$ are $k-$independent and are values at this point $\tilde{K}$. Thus, these two quantities are now determined via the wave equation

$$\frac{\partial^2 E_{0y}(z)}{\partial z^2} - \tilde{K}^2 E_{0y}(z) = -\bar{\varepsilon}(z)\frac{\omega_{\tilde{K}}^2}{c^2}E_{0y}(z). \tag{S8}$$

Combining this equation with the wave equation for electromagnetic waves in the grating, we arrive at

$$\left[\bar{\varepsilon}(z)\frac{\omega^2 - \omega_{\tilde{K}}^2}{c^2} + \tilde{K}^2 - (k_x + K_n)^2\right] E_{0y}(z)C_n = -\frac{\omega^2}{c^2}\left[E_{0y}(z)\sum_{l\in\mathcal{B}, l\neq n}\xi_{n-l}(z)C_l + \sum_{u\in\mathcal{O}}\xi_{n-u}(z)C_u(z)\right]. \tag{S9}$$

Then, multiplying both sides by $E_{0y}^*(z)$ and taking integration over z yields

$$\left\{\omega^2 - \omega_{\tilde{K}}^2 + \frac{c^2}{\bar{n}_0^2}\left[\tilde{K}^2 - (k_x + K_n)^2\right]\right\}C_n = -\alpha\omega^2\sum_{l\in\mathcal{B}, l\neq n}\xi_{n-l}C_l - \omega^2\sum_{u\in\mathcal{O}}\xi_{n-u}\int_{-\infty}^{+\infty}dz E_{0y}^*(z)C_u(z)f_{\mathrm{e}}(z) \tag{S10}$$

with $\bar{n}_0^2 = \int_{-\infty}^{+\infty}|E_{0y}(z)|^2\,\bar{\varepsilon}(z)dz$ and $\alpha = \int_{-\infty}^{+\infty}|E_{0y}(z)|^2\,f_{\mathrm{e}}(z)dz$. We choose the point of interest $\tilde{K} = K_1/2$, notate $\omega_{\tilde{K}} = \omega_0$, and consider two basic waves corresponding to $n = 0$ and $n = -1$, which gives two coupled equations

$$\left\{\omega^2 - \omega_0^2 + \frac{c^2}{\bar{n}_0^2}\left[\frac{K_1^2}{4} - (k_x + K_{-1})^2\right]\right\}C_{-1} = -\alpha\omega^2\xi_{-1}C_0 - \omega^2\sum_{u\neq 0,-1}\xi_{-1-u}\int_{-\infty}^{+\infty}dz E_{0y}^*(z)C_u(z)f_{\mathrm{e}}(z), \tag{S11a}$$

$$\left\{\omega^2 - \omega_0^2 + \frac{c^2}{\bar{n}_0^2}\left(\frac{K_1^2}{4} - k_x^2\right)\right\}C_0 = -\alpha\omega^2\xi_1 C_{-1} - \omega^2\sum_{u\neq 0,-1}\xi_{-u}\int_{-\infty}^{+\infty}dz E_{0y}^*(z)C_u(z)f_{\mathrm{e}}(z). \tag{S11b}$$

Define $k = k_x - K_1/2$ and neglect the interaction with higher-order modes ($u \neq 0, -1$), we obtain

$$\left\{\omega^2 - \omega_0^2 + \frac{c^2}{\bar{n}_0^2}\left[\frac{K_1^2}{4} - \left(k - \frac{K_1}{2}\right)^2\right]\right\}C_{-1} = -\alpha\omega^2\xi_{-1}C_0 \Rightarrow \left[\omega^2 - \omega_0^2 - \frac{c^2}{\bar{n}_0^2}\left(k^2 - K_1 k\right)\right]C_{-1} = -\alpha\omega^2\xi_{-1}C_0, \tag{S12a}$$

$$\left\{\omega^2 - \omega_0^2 + \frac{c^2}{\bar{n}_0^2}\left[\frac{K_1^2}{4} - \left(k + \frac{K_1}{2}\right)^2\right]\right\}C_0 = -\alpha\omega^2\xi_1 C_{-1} \Rightarrow \left[\omega^2 - \omega_0^2 - \frac{c^2}{\bar{n}_0^2}\left(k^2 + K_1 k\right)\right]C_0 = -\alpha\omega^2\xi_1 C_{-1}. \tag{S12b}$$

If the periodic modulation in the dielectric function of the grating is sufficiently weak, we can considerably simplify these equations by assuming that $\boxed{|\omega - \omega_0| \ll \omega_0}$ and neglecting the term of $(\omega - \omega_0)\xi_{\pm 1}$. The equations then become

$$\left[\omega - \omega_0 - \frac{c^2}{2\bar{n}_0^2\omega_0}\left(k^2 - K_1 k\right)\right]C_{-1} \approx -\frac{\alpha\omega_0\xi_{-1}}{2}C_0, \tag{S13a}$$

$$\left[\omega - \omega_0 - \frac{c^2}{2\bar{n}_0^2\omega_0}\left(k^2 + K_1 k\right)\right]C_0 \approx -\frac{\alpha\omega_0\xi_1}{2}C_{-1}. \tag{S13b}$$

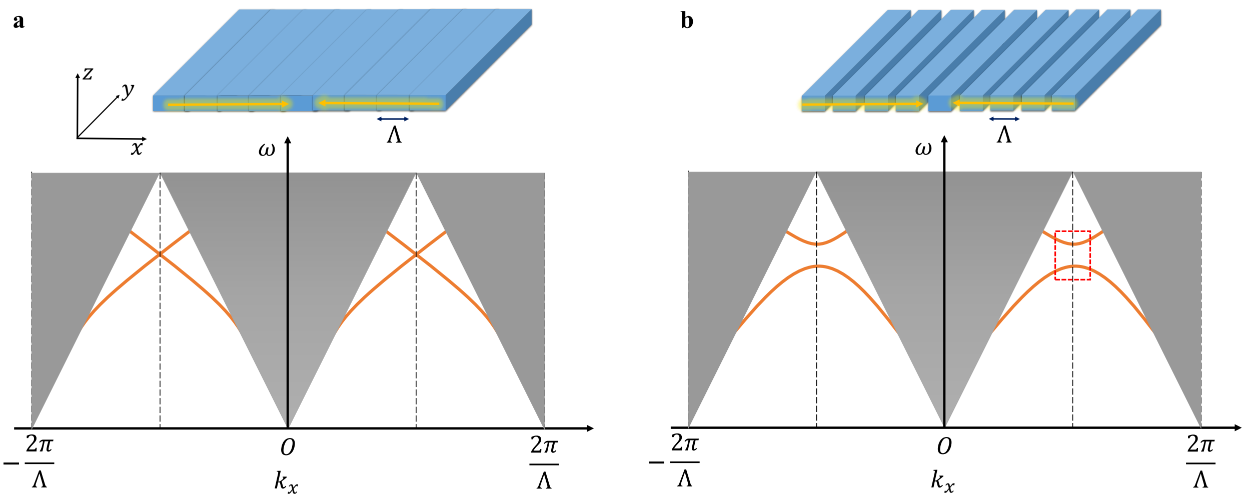

Figure S1: Dispersion of the lowest guided mode in (a) a homogeneous waveguide with infinitesimal periodic modulation of dielectric, and (b) a dielectric grating. The red dashed box indicates the region where the effective model is valid.

Rewriting these two equations in matrix form gives us the effective model of a single grating

$$\omega_0 + \frac{c^2}{2\bar{n}_0^2\omega_0}k^2 + \begin{pmatrix} \bar{v}k & U \\ U^* & -\bar{v}k \end{pmatrix}\begin{pmatrix} C_0 \\ C_{-1} \end{pmatrix} = \omega \begin{pmatrix} C_0 \\ C_{-1} \end{pmatrix} \tag{S14}$$

with $\bar{v} = \dfrac{\pi c^2}{\bar{n}_0^2\omega_0\Lambda}$ and $U = -\dfrac{\alpha\omega_0\xi_1}{2}$. Owing to the inversion symmetry along the x direction of the dielectric function, we have $U = U^*$. For simplicity, we neglect the k-quadratic term as it has no effect on the topological properties of the spectrum | the wave equation can thus be written as an eigen-equation $H_{\text{mono}}(k)\Psi(k) = \omega_k\Psi(k)$ with the operator

$$H(k) = \omega_0 + \begin{pmatrix} \bar{v}k & U \\ U & -\bar{v}k \end{pmatrix} \tag{S15}$$

termed the Hamiltonian and $\Psi(k) = \begin{pmatrix} C_0 & C_{-1} \end{pmatrix}^T$.

The basis functions of this Hamiltonian consists of two plane waves $\varphi_+ = E_{0y}(z)e^{ik_x x} = E_{0y}(z)e^{i(k+\pi/\Lambda)x}$ and $\varphi_- = E_{0y}(z)e^{i(k_x-2\pi/\Lambda)x} = E_{0y}(z)e^{i(k-\pi/\Lambda)x}$. The electric field is hence given by

$$E_y(\mathbf{r}) = E_{0y}(z)\left[C_0 e^{i(k+\pi/\Lambda)x} + C_{-1}e^{i(k-\pi/\Lambda)x}\right]. \tag{S16}$$

This effective model can be interpreted as depicted in Fig. S1. In a homogeneous slab waveguide with infinitesimal periodic modulation of dielectric constant, two counter-propagating guided modes have group velocity $\bar{v}$ when their wave numbers are around $k_x = \pi/\Lambda$ (X point). In the presence of periodic corrugation, these modes diffract and couple with each other with strength U. If the grating translates along the x axis by δ, its dielectric function is given by $\varepsilon'(x) = \varepsilon(x-\delta)$. While the zeroth Fourier component ξ_0' remains unchanged, the first component varies as follows

$$\begin{aligned}\xi_1' = \int_{-\infty}^{+\infty} dx\,[\varepsilon'(x) - 1]\,e^{-i2\pi x/\Lambda} &= \int_{-\infty}^{+\infty} dx\,[\varepsilon(x-\delta) - 1]e^{-i2\pi x/\Lambda} \\ &= \int_{-\infty}^{+\infty} dx\,[\varepsilon(x) - 1]\,e^{-i2\pi(x+\delta)/\Lambda} = \xi_1 e^{-i2\pi\delta/\Lambda}.\end{aligned} \tag{S17}$$

As a result, we arrive at the substitution $U \to Ue^{-i2\pi\delta/\Lambda}$.

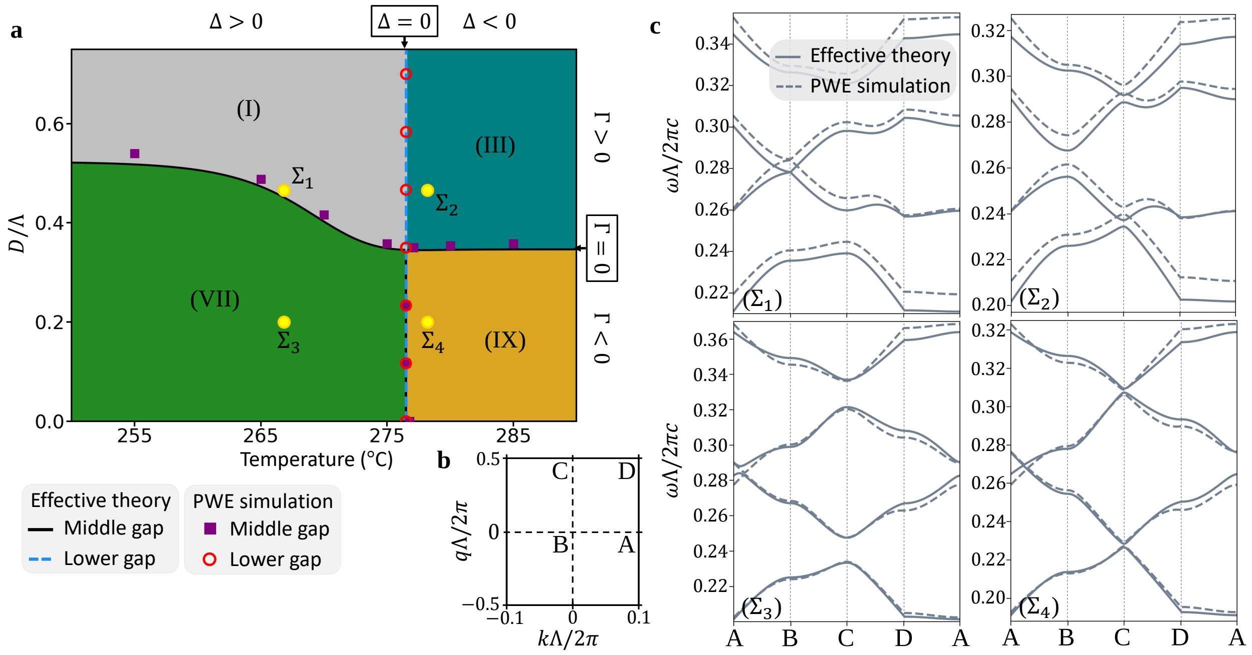

Figure S2: Comparison between the bulk band structure obtained from PWE simulations and the one calculated by the effective theory. (a) The phase diagram presented in the main text. (b) The contour ABCDA in momentum space that is used to plot the band structures. (c) The four band structures at the four Σ-points in (a).

For the bilayer grating, we can follow a similar procedure to obtain the effective Hamiltonian. However, for simplicity, we treat the problem phenomenologically by assuming that the co-propagating modes in the two layers couple with each other only through the evanescent field. Other interlayer coupling mechanisms are negligible. With the evanescent coupling strength notated V, we achieve the effective Hamiltonian shown in the main text

$$H(k,\delta) = \begin{pmatrix} \omega_1 + v_1 k & U_1 e^{-i2\pi\frac{\delta}{\Lambda}} & V & 0 \\ U_1 e^{i2\pi\frac{\delta}{\Lambda}} & \omega_1 - v_1 k & 0 & V \\ V & 0 & \omega_2 + v_2 k & U_2 \\ 0 & V & U_2 & \omega_2 - v_2 k \end{pmatrix}, \tag{S18}$$

whose eigenvalues $\omega(k,q)$ are the frequencies of the four lowest guided modes in the vicinity of the X-point

$$H(k,\delta)\begin{pmatrix} C_0^u \\ C_{-1}^u \\ C_0^l \\ C_{-1}^l \end{pmatrix} = \omega(k,q)\begin{pmatrix} C_0^u \\ C_{-1}^u \\ C_0^l \\ C_{-1}^l \end{pmatrix}. \tag{S19}$$

Here, the indices u and l denote the upper grating and lower grating, respectively. The electric field of these TE modes is given by

$$E_y(\mathbf{r}) = E_{0y}^u(z)\left[C_0^u e^{i(k+\pi/\Lambda)x} + C_{-1}^u e^{i(k-\pi/\Lambda)x}\right] + E_{0y}^l(z)\left[C_0^l e^{i(k+\pi/\Lambda)x} + C_{-1}^l e^{i(k-\pi/\Lambda)x}\right]. \tag{S20}$$

For short notations, we write $E_y(\mathbf{r}) = \sum_{\nu=1}^{4}\Psi_\nu\varphi_\nu$ for $\Psi_\nu \in \{C_0^u, C_{-1}^u, C_0^l, C_{-1}^l\}$ and

$$\varphi_\nu \in \left\{E_{0y}^u(z)e^{i(k+\pi/\Lambda)x}, E_{0y}^u(z)e^{i(k-\pi/\Lambda)x}, E_{0y}^l(z)e^{i(k+\pi/\Lambda)x}, E_{0y}^l(z)e^{i(k-\pi/\Lambda)x}\right\}.$$

The parameters of the Hamiltonian can be retrieved through comparison with rigorous simulations at some special points, as exemplified in Fig. S8 for silicon gratings. With these parameters, the four frequency

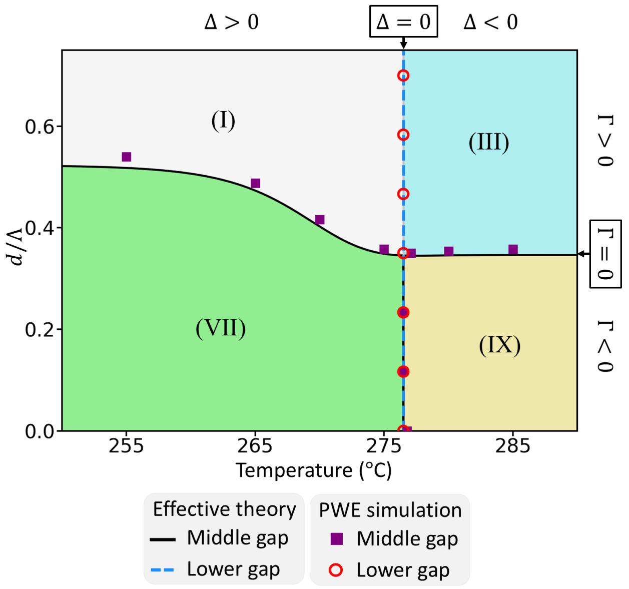

Figure S3: **Phase diagram controlled by varying the temperature and interlayer distance.** The bilayer comprises an antimony trisulfide grating and an amorphous silicon grating. The solid black (dashed blue) line denotes where the middle (lower) gap closes within the effective theory. The purple squares (red circles) correspond to similar points obtained through PWE simulations.

bands can be straightforwardly obtained by exact diagonalization and are shown in Fig. S9. The effective model indeed agrees excellently with Finite-Difference Time-Domain (FDTD) simulations. We carry out the same fitting process for our setup in the main text. From Figs. S2(c), we see that the effective model again agrees with PWE simulations. We note that in case (Σ_3) of Fig. S2(c), the two middle bands obtained from PWE simulation has a gap at the AB segment which is small compared to the one given by the effective theory.

Dynamic Control of Topological Phases via Phase-Change Material

The topological phases presented previously can be observed in a single sample by means of PCMs[1–4]. In this work, we incorporate into the bilayer photonic crystal the PCM antimony trisulfide (Sb_2S_3), which is well known for its ultralow losses across the visible and near-infrared wavelengths in both crystalline and amorphous phases[5,6], and its exceptional tunability[7,8]. Its phase can be changed reversibly, either by heating the entire sample or by shining laser pulses at a specific spot.

In our bilayer system, as the intralayer coupling strength depends on the refractive index, we vary Δ by switching the material's phase between amorphous and crystalline. We demonstrate this idea by designing a bilayer system of an amorphous silicon (refractive index 3.15) grating and an Sb_2S_3 grating. The geometrical parameters are $w_1 = w_2 = 0.8\Lambda$ and $h_1 = h_2 = 0.37\Lambda$. The refractive index of Sb_2S_3 increases continuously from 2.73 to 3.26 when it transitions from amorphous to crystalline phase. This transition is complete when the temperature is raised above 280°C[9]. Amorphization can be achieved by heating the crystalline Sb_2S_3 above its melting temperature and then rapidly quenching. An on-chip reversible transition of PCM can be obtained using a state of the art microheater[7,10–13]. Here, by fitting the experimental data describing the temperature-dependent refractive index of Sb_2S_3 during its crystallization[9], we obtained a function analogous to the logistic function

$$n_{\mathrm{Sb_2S_3}} = A + B\left\{1 - \frac{1}{1 + 0.5\left[e^{\alpha(T-T_0)} + e^{\beta(T-T_0)}\right]}\right\}$$

with $A = 2.732738$, $B = 0.530212$, $\alpha = 0.470\,62\,\mathrm{K}^{-1}$, $\beta = 0.233\,60\,\mathrm{K}^{-1}$, and $T_0 = 273\,\mathrm{K}$. The PWE

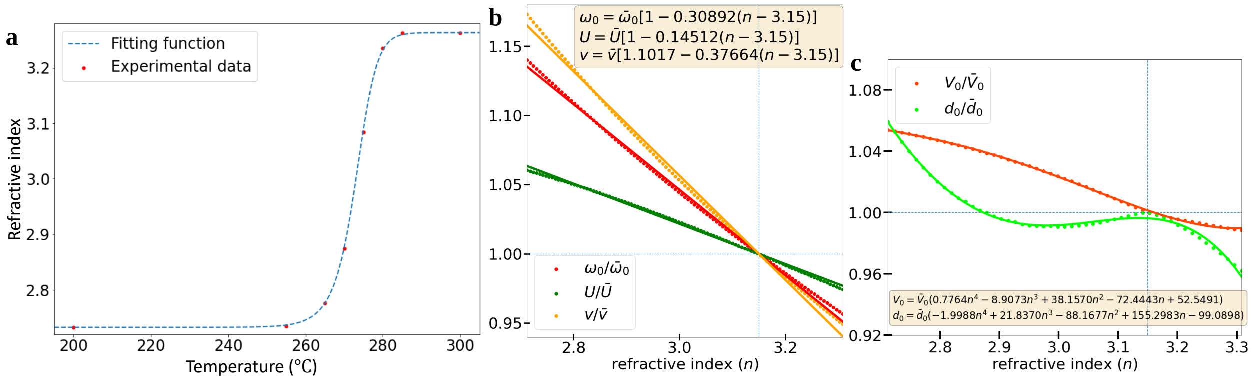

Figure S4: (a) The dependence of Sb_2S_3 refractive index on temperature. (b) Variation of model parameters of a single grating ($L = 0.8\Lambda$ and $H = 0.37\Lambda$) with respect to the refractive index. (c) Variation of interlayer coupling strength and decay length with respect to the refractive index.

simulations are run accordingly using this function. The dependence of parameters ω_2, v_2, and U_2 of the PCM grating and the interlayer coupling V on temperature is achieved by fitting the effective model of the monolayer and bilayer lattices with PWE results. The experimental data of Sb_2S_3 and the temperature dependence of the effective model's parameters are presented in the SM.

On the other hand, to alter Γ, the interlayer distance can be adjusted dynamically using on-chip MEMSs[14,15]. Such a combination of thermal and mechanical control of the bilayer grating allows us to achieve the complete phase diagram shown in Fig. S3 on a single sample, corresponding to Sb_2S_3 crystallization. The four regions are associated with the four gapped states, i.e., the four frequency bands are disconnected, while their borders correspond to the gapless ones. These gap-closing lines match well with the results obtained from the PWE simulation using MIT Photonic Bands package[16].

The phase diagram is general and can be achieved with other dielectric materials as long as the geometrical parameters are appropriate. As considered above, despite replacing amorphous silicon with indium phosphide (InP), whose refractive index is 3.17, all the topological phases remain. This generality implies possible optimization of the bilayer grating for specific properties, such as the spectral gap or the quality factor of the heterostructure.

Temperature Dependence of Antimony Trisulfide

Antimony trisulfide (Sb_2S_3) is an ultra-low loss phase-change material. The dependence of its refractive index on temperature for progressive crystallization is presented in the table below, which was provided by the authors of Ref. [9]

Refractive index for wavelength 1500 nm								
Temperature (°C)	200	255	265	270	275	280	285	300
Refractive index	2.732738	2.735401	2.776021	2.87417	3.084485	3.235228	3.26294	3.26295

The continuous change in refractive index arises from gradual partial crystallization. The apparition of nucleation sites of Sb_2S_3 is reported to have perfectly random and homogeneous distribution[9]. We fit these data with a function analogous to the logistic function

$$n_{\mathrm{Sb_2S_3}} = A + B\left\{1 - \frac{1}{1 + 0.5\left[e^{\alpha(T-T_0)} + e^{\beta(T-T_0)}\right]}\right\} \tag{S21}$$

with $A = 2.732738$, $B = 0.530212$, $\alpha = 0.470\,62\,\mathrm{K}^{-1}$, $\beta = 0.233\,60\,\mathrm{K}^{-1}$, and $T_0 = 273\,\mathrm{K}$, as shown in Fig. S4(a).

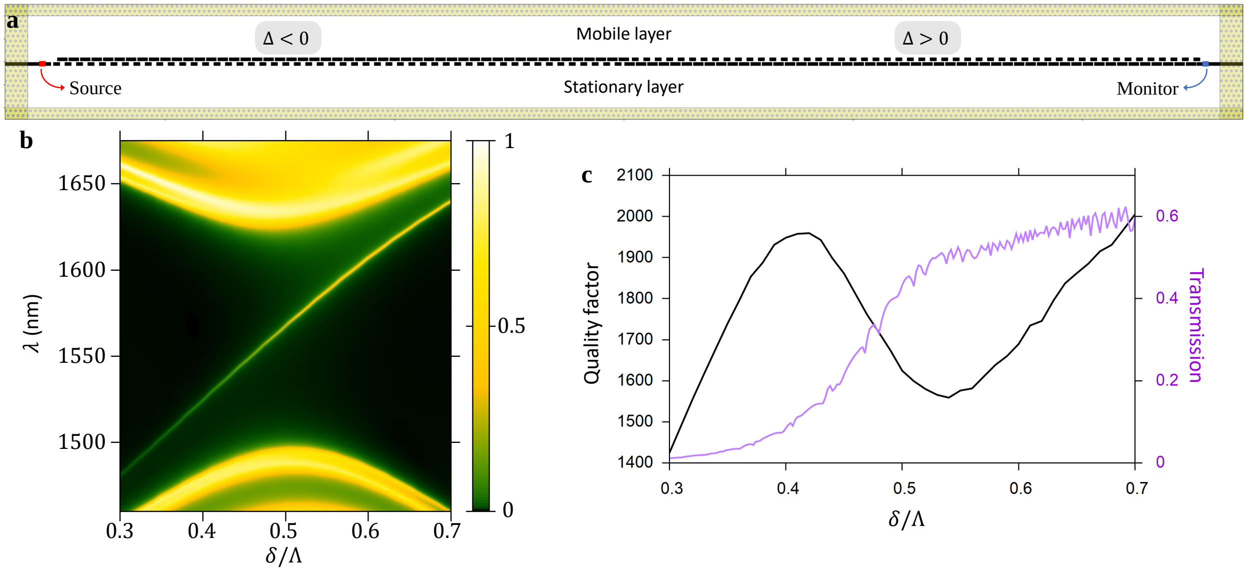

Figure S5: Photonic junction as a light filter. (a) FDTD computational cell of the heterojunction, which consists of two bilayer gratings with opposite values of Δ. The cell is surrounded by phase-matching layers that dissipates the electromagnetic field. (b) The guided transmission spectrum of the system as the upper grating translates gradually. (c) The quality factor and transmission coefficient of the interface mode.

To obtain the phase diagram in Fig. 7, we retrieve the dependence of model parameters on the refractive index of the Sb_2S_3 grating. First, we consider a single Sb_2S_3 grating with $L = 0.8\Lambda$ and $H = 0.37\Lambda$ and see how the model parameters vary. The simulation data are obtained by PWE with MPB package. We crudely fit the data with linear functions in comparison with parameters ($\bar{\omega}_0, \bar{U}$, and $\bar{v}$) of an identical grating made of amorphous silicon (aSi), as shown in Fig. S4(b). Then, we examine a bilayer of Sb_2S_3 grating and aSi grating with the same geometrical parameters. The interlayer interaction between them is approximated as $V(D) = V_0 \exp(-D/d_0)$. The dependence of V_0 and d_0 is fit with polynomial functions – see Fig. S4(c). Here, $\bar{V}_0$ and $\bar{d}_0$ correspond to the case when Sb_2S_3 refractive index is 3.15, the same as amorphous silicon.

The parameters are

$$\frac{\bar{\omega}_0\Lambda}{2\pi c^2} = 0.26304, \qquad \frac{\bar{U}\Lambda}{2\pi c^2} = 0.02181, \qquad \bar{v} = 0.36222c, \qquad \frac{\bar{V}_0\Lambda}{2\pi c^2} = 0.06067, \qquad \bar{d}_0 = 0.35599\Lambda.$$

Guided Transmission for Broadband Filtering

In this section, we demonstrate that a heterojunction of the bilayer grating can be used as a tunable filter.

Design

The design of the light filter is shown in Fig. S5(a), which is a photonic heterojunction composed of two aligned bilayer gratings on two sides. Each bilayer system consists of two silicon gratings (refractive index 3.5) with identical thickness $H = 0.3\Lambda$ and different values of width: $L_1 = 0.9\Lambda$ and $L_2 = 0.58\Lambda$. The interlayer separation between the two gratings is 0.1Λ. The two sides of the junction share the same structure but the two gratings are swapped, which reverses the sign of Δ across the junction. The lower layer is attached stock-still to a source and a monitor, both of which are embedded in a silicon padding block, while the upper layer is mobile and used as a “tunable knob".

The number of lattice periods per side of the junction is 20. The source emits a pulse with Gaussian shape in frequency, centering at $0.218c/\Lambda$ and of width $0.03c/\Lambda$. The FDTD simulations are carried out using MEEP.

Guided transmission

From the main text, we know that a localized interface mode exists in this junction due to the topological phase transition, and it is chiral along the synthetic dimension δ. Importantly, as seen in Fig. 4D, this mode exponentially decays into the bulk | we can thus excite this mode by putting a source in its decaying tail. The upper layer is translated along the x direction to tune the frequency of the edge mode by varying δ.

The transmission spectrum for various values of δ is shown in Fig. S5(b) with lattice period $\Lambda = 340$ nm. We see that the system in this case can filter a wide range of wavelength, from approximately 1450 nm to 1650 nm[2]. The quality factor of the transmitted signal is shown in Fig. S5(c), which is above 1000, implying the excellent performance of light filtering even for a wide band gap. This quality factor becomes greater when the system size gets larger, i.e., larger N, as we have seen in Fig. 4E. However, as the source is further away from the interface, the transmitted signal decreases exponentially. We can optimize the system size to obtain the desirable output as decreasing the size decreases the quality factor but increases the transmission intensity. Additionally, Fig. S5(c) also presents the transmission of the edge mode, which depends strongly on the shift δ.

Advantages

Besides being a high-quality filter over a wide range of wavelength, this photonic junction is also a filter robust against disorders and defects. Since the chiral edge mode is topologically protected in the synthetic momentum space, it consistently traverses the spectral gap even in the presence of perturbations[17]. Consequently, with the relative displacement δ being dynamically adjustable, one can always tune the edge mode to achieve the desirable wavelength.

Finite-Difference Time-Domain Simulations

All the FDTD simulations in this work are carried out by either the MEEP package[18] or the commercial software Lumerical.

Spectrum – The spectra shown in Figs. 4(b) and 4(c) of the photonic heterojunction are obtained from Lumerical FDTD simulations. A dielectric photonic junction is constructed following the geometry depicted in Fig. 4(a) with its interface lying at the center of the computational cell. The refractive indices of the upper and lower gratings are 3.17 and 2.73, respectively. In this linear regime, the parameters scale with the lattice constant Λ, so we set $\Lambda = 1\,\mu$m for simplicity. The total number of periods is 400, i.e., the length of the heterojunction is 400 µm. The 2D computational cell is enclosed in standard phase-matching layers. The mesh for finite-difference calculation has the maximum mesh step 0.02 µm along the x direction and 67 mesh cells per wavelength along the y direction (i.e., z direction in Results). The electromagnetic modes of the system are excited by 20 electric dipoles randomly distributed in the bilayer within a range of 160 µm around the interface. The dipoles are aligned along the z axis ($\theta = 0$), have random phases and random angle with respect to the x axis. Each of them emits a broadband pulse with frequency ranging from 69 THz to 74 THz. The simulation runs for 70 ps at 300 K. All signals are recorded and analyzed by 20 time monitors randomly distributed in the system within a range of 240 µm around the interface. We note that the spectra in Fig. 4 have a few discrete patterns. They are numerical artifacts resulting from the dielectric gratings crossing a mesh line.

Quality factor – The quality factor of the edge mode is computed using MEEP and Lumerical simulations, with both methods yielding comparable results. In the MEEP simulations, a dielectric photonic junction is constructed similar to that in Lumerical. A single point source, emitting a Gaussian pulse with a frequency width of $\Delta f = 0.002(c/\Lambda)$, is randomly embedded in a dielectric rod at the interface. The central frequency of this optical pulse follows a straight trajectory along the chiral edge mode, $f_{\text{center}} = (0.06\delta + 0.2086)(c/\Lambda)$. The source excites modes with an electric field parallel to the dielectric rod. A monitor is placed inside another dielectric rod at the junction interface to analyze the response for 10^4 time units after the source has turned off. The 2D computational cell has a resolution of 32 and dimensions

[2] As the spectral gap has not been optimized in this example, the range of filtering wavelength can certainly be further increased.

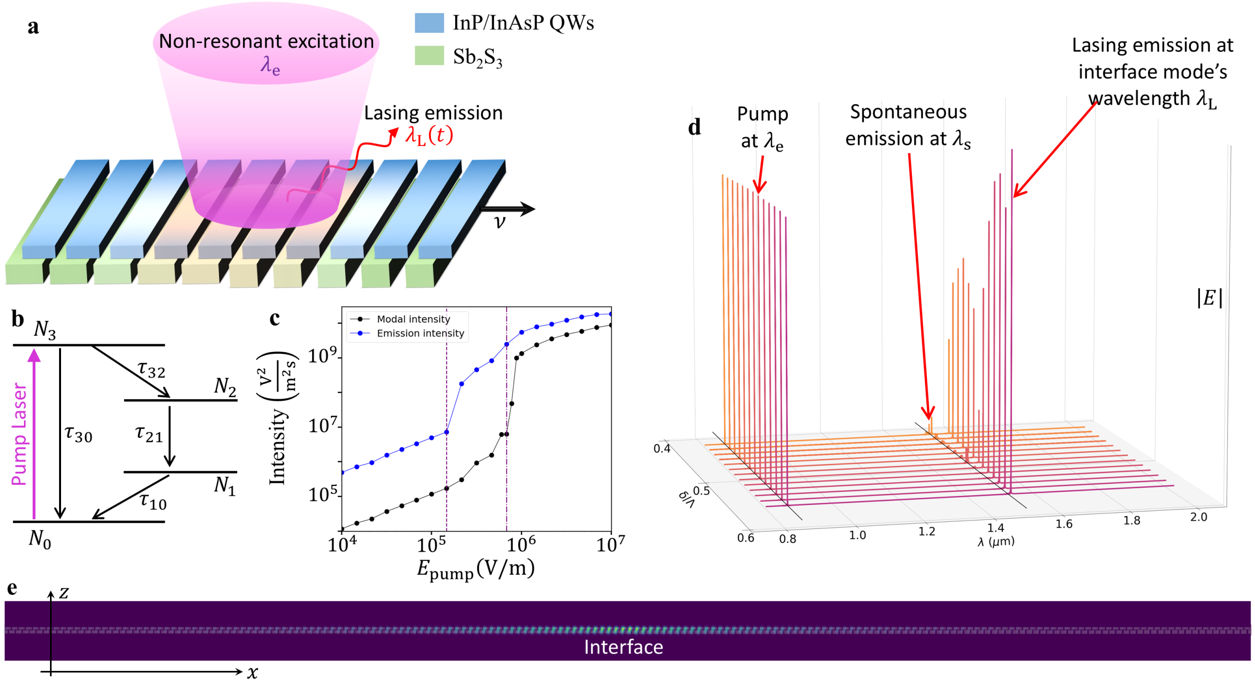

Figure S6: (a) The heterojunction of bilayer photonic crystal where the upper layer is made of gain material InAsP/InP moves slowly with velocity ν. The heterojunction is continuously pumped by a non-resonant spatially Gaussian source of wavelength λ_e and achieves lasing action at λ_L. (b) Schematic diagram of the four-level two-electron model describing the gain material. (c) Emission and modal intensities with respect to the pump field strength when $\delta = 0.5\Lambda$. The dashed line indicates where population inversion starts to take place while the dashed dotted line indicates the lasing threshold. (d) Complete spectrum detected at the monitors and (e) the electric field profile of the lasing mode at $\delta = 0.5\Lambda$ when $E_{\text{pump}} = 1.3 \times 10^6\ \text{V m}^{-1}$.

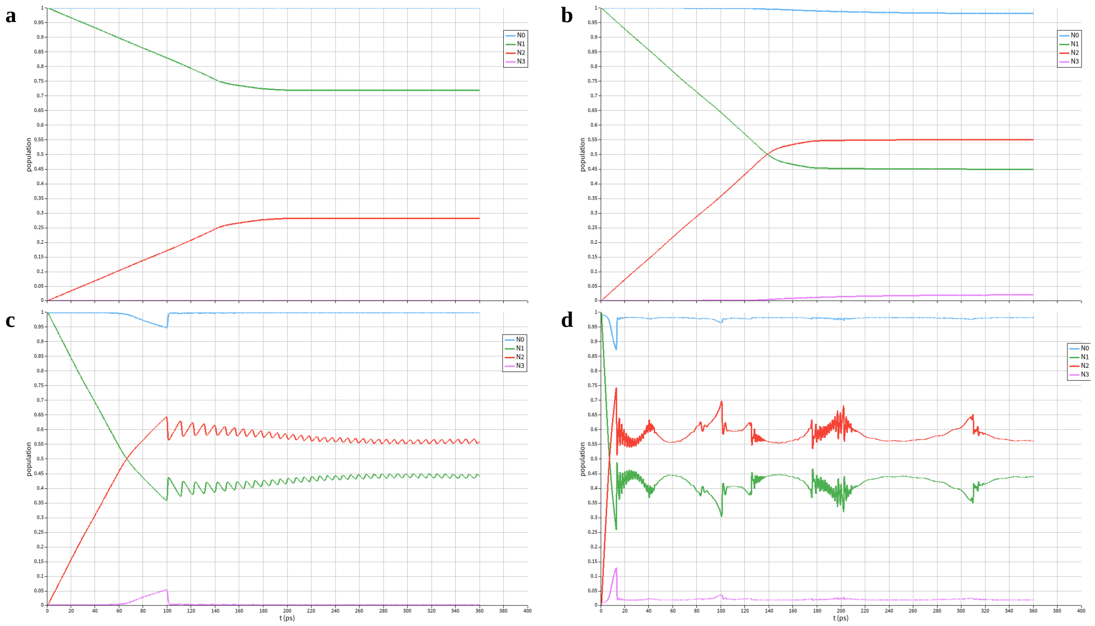

Figure S7: Electron population density probability of the four levels at different pump field strengths: (a) $E_{\text{pump}} = 10^5$ V/m, (b) $E_{\text{pump}} = 146780$ V/m, (c) $E_{\text{pump}} = 215440$ V/m, (d) $E_{\text{pump}} = 316230$ V/m.

of $(N + 7, 26)$, where N is the number of periods on each side. The boundary layers perpendicular to the y-axis are phase matching layers of thickness 2, while those normal to the x-axis are adiabatic absorbers of thickness 7. The periodic lattices submerge into the absorbers.

Lasing simulation – For lasing simulations in Lumerical FDTD, the heterojunction is constructed similarly but we use a realistic geometry with $\Lambda = 366\,\text{nm}$ since the calculations are nonlinear. The lower grating is still a dielectric with the refractive index 2.73 while the upper grating now is modeled by a four-level two-electron material[19], akin to what is depicted in Fig. 5(b). In this gain material, the transition wavelengths are $\lambda_{\text{s}} = 1.5\,\mu\text{m}$ and $\lambda_{\text{e}} = 0.85\,\mu\text{m}$, the damping coefficients are $\gamma_{\text{a}} = \gamma_{\text{b}} = 10^{13}$ Hz, the lifetimes of different decay channels are $t_{30} = t_{21} = 3 \times 10^{-10}$ s and $t_{32} = t_{10} = 10^{-13}$ s, and the electron population density is $1 \times 10^{23}\,\text{m}^{-3}$. The heterojunction is continuously pumped by a spatial-Gaussian beam with wavelength λ_{e} and waist radius $2\,\mu\text{m}$, located $2.2\,\mu\text{m}$ above the system. The signals are recorded and analyzed by 10 time monitors located $0.5\,\mu\text{m}$ below the system. The heterojunction in these simulations has 300 periods, corresponding to a length of approximately $110\,\mu\text{m}$. The simulations run for 360 ps at 300 K. The mesh for finite-difference calculation has the maximum mesh step $0.006\,\mu\text{m}$ along the x direction and 60 mesh cells per wavelength along the y direction.

In our lasing simulation with Lumerical FDTD - the setup is re-sketched in Fig. S6(a), the gain material is described by a 4-level 2-electron model, which is depicted in Fig. S6(b). In this model, the electron transitions are treated as two coupled dipole oscillators, one corresponds to levels 1 and 2 while the other is associated with levels 0 and 3. These transitions are governed by the coupled rate equations and the Pauli exclusion principle, and they are solved self-consistently. At $t = 0$, the electron populations are $N_0 = N_1 = 1$ and $N_2 = N_3 = 0$. Other parameters are given in the Materials and Methods.

We remark on Fig. 5(e) of the main text, where we observe a slight discontinuity in the emission signals along the chiral interface mode and a corresponding dip in intensity. This stems from the coupling between the central interface mode and an unphysical interface mode at the boundary of the computational cell, caused by the finite-size effects of the structure and the finite thickness of the absorbing layers. Such in-plane leakage of the interface mode leads to the surge at $\delta = 0.5\Lambda$ in the Q-factor shown in Fig. 4(e). On the other hand, we note that the signal detected by the monitors depends not only on the Q-factor of the cavity but also on the positions of the source and the monitors. In our current simulation, the monitors are

located directly below the interface, which do not capture the entire lasing emission. For instance, if the angular dependence of the lasing emission varies with respect to the lateral shift δ, the signal's intensity will vary accordingly, independent of the Q-factor. Hence, the variation of the emission intensity with respect to δ detected at the monitors is a combined function of the Q-factor and the spatial configuration of the source and monitors.

When examining the lasing action with respect to the field strength of the pump at $\delta = 0.5\Lambda$, there are two ways to present the emission signal. The first one is to compute the lasing modal intensity, which is shown in the main text. Here, we focus solely on the intensity of light at the lasing wavelength. The second way is to compute the emission intensity, which is also defined by $\frac{1}{2\pi}\int d\omega |E|^2$ but with the integration taken over the frequency range ω encompassing all the emission peaks. Both of these quantities are shown in Fig. S6(c) against the pump field strength. On the one hand, we see that the emission intensity depicts nicely where the population inversion (between level 1 and 2) starts to take place, which is $E_{\text{inv}} \approx 1.5 \times 10^5$ V/m. Between E_{inv} and E_{thres} is where amplified spontaneous emission dominates, i.e., population inversion is present with no distinguishable signal at the resonant wavelength. This process does not involve any resonator, e.g., a cavity, and has a broad bandwidth in its emission spectrum, centered at $\lambda_{\text{s}} = 1500$ nm. It is expected to take place here owing to the large volume of gain material. The electron populations at some values of field strengths around this transition are shown in Fig. S7. On the other hand, the modal intensity illustrates well the lasing threshold $E_{\text{thres}} \approx 7 \times 10^5$ V/m where the lasing peak starts to appear. As shown in the main text and Fig. S6(d) for field strength $E_{\text{pump}} = 1.3 \times 10^6\,\text{V}\,\text{m}^{-1}$ above the threshold, when population inversion as well as steady state are achieved, the lasing mode appears as a sharp peak at the wavelength λ_{L} of the localized interface mode. It varies linearly against δ due to the chiral nature, different from the signals from the pumping source and spontaneous emission, whose wavelengths always center at λ_{e} and λ_{s}, respectively – see Fig. S6(d). We further confirm that this signal indeed comes from the topological interface mode by plotting its field profile in Fig. S6(e), which localizes at the interface, in agreement with the interface mode's profile.

Edge State from Effective Model

In Figure 5D of the main text, we use the effective model to compute the chiral edge mode within the synthetic space, which serves as a guide for the simulation results. The method for this calculation is detailed in the Supplemental Material of Ref. [17].

Captions of Supplemental Videos

Caption for Movie S1

Particle pumping and trapping in bipartite potential. Time evolution of two periodic potentials of the same periodicity where $U_1(x,t)$ moves slowly and $U_2(x) = 1.5\sin(2\pi x/\Lambda)$ is stationary, with $U_1(x,t) = 2.2\sin(2\pi x/\Lambda - 2\pi\nu t/\Lambda)$ (left) and $U_1(x,t) = 1.2\sin(2\pi x/\Lambda - 2\pi\nu t/\Lambda)$ (right). The particles (denoted by black dots) can either be transported by $U_1(x,t)$ to the next unit cell (pumping), or be pulled back by $U_2(x)$ to their original positions (trapping).

Caption for Movie S2

Optical pumping and trapping in bilayer photonic lattice. Time evolution of the electric field profile of the lowest mode at $k = 0$ at various moments in the sliding bilayer photonic lattice. The two configurations of the lattice are associated with two regimes: topological pumping and trapping. The electric field is computed using the plane-wave expansion method implemented in the MIT photonic bands package.

Supplemental Figures

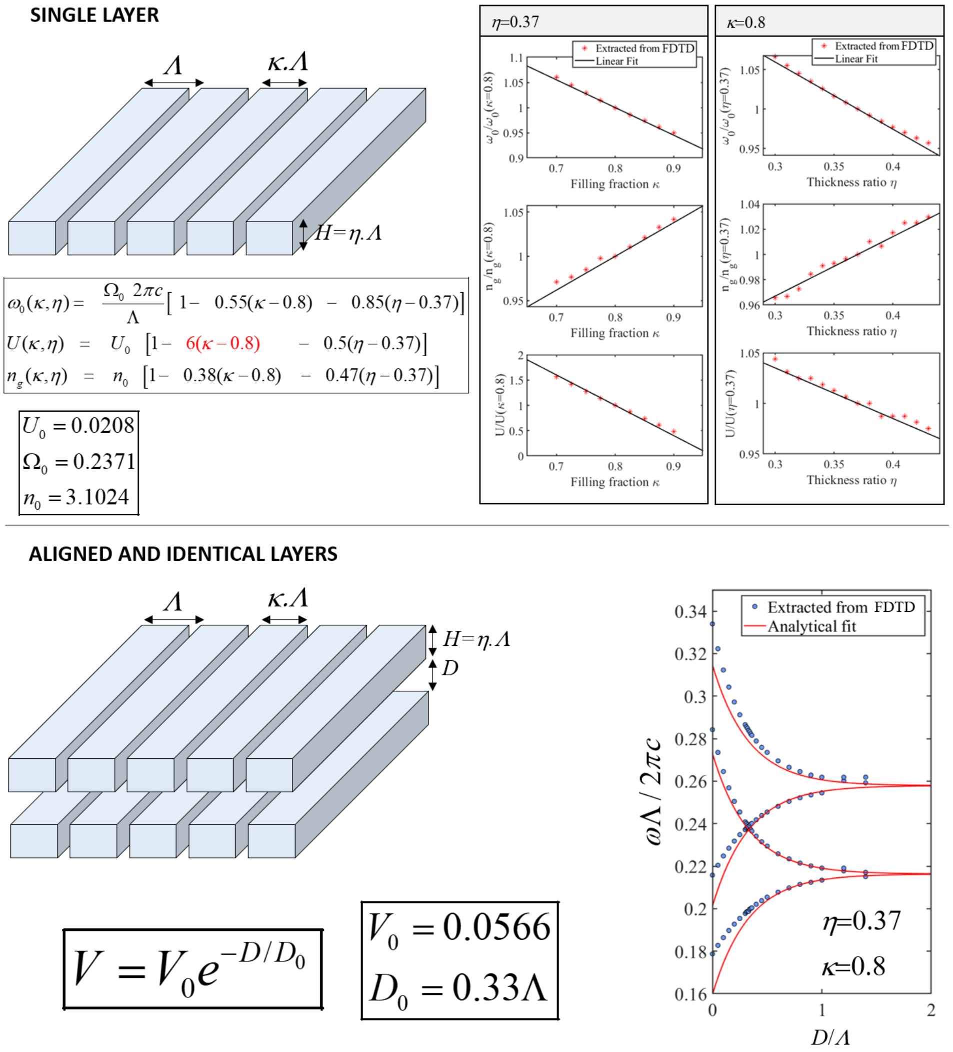

Figure S8: Parameter retrievals for the effective Hamiltonian from FDTD simulations. Upper Panels: the simulations for a single layer grating are used to obtain the dependence of ω_0, U and n_g as functions of the filling fraction κ and thickness ratio η. These parameters are extracted by fitting the two band dispersion with the Hamiltonian (S15). Lower Panels: the simulations for a bilayer of aligned and identical gratings at $k = 0$ are used to obtain the dependence of V as functions of the distance D separating the two layers. The exponential decay law of V is obtained by fitting the four band edges $\omega_0 + U + V$, $\omega_0 + U - V$, $\omega_0 - U + V$, and $\omega_0 - U - V$ at varying distance D. Here, the values of U and ω_0 are already known from the retrievals from the single layer simulations.

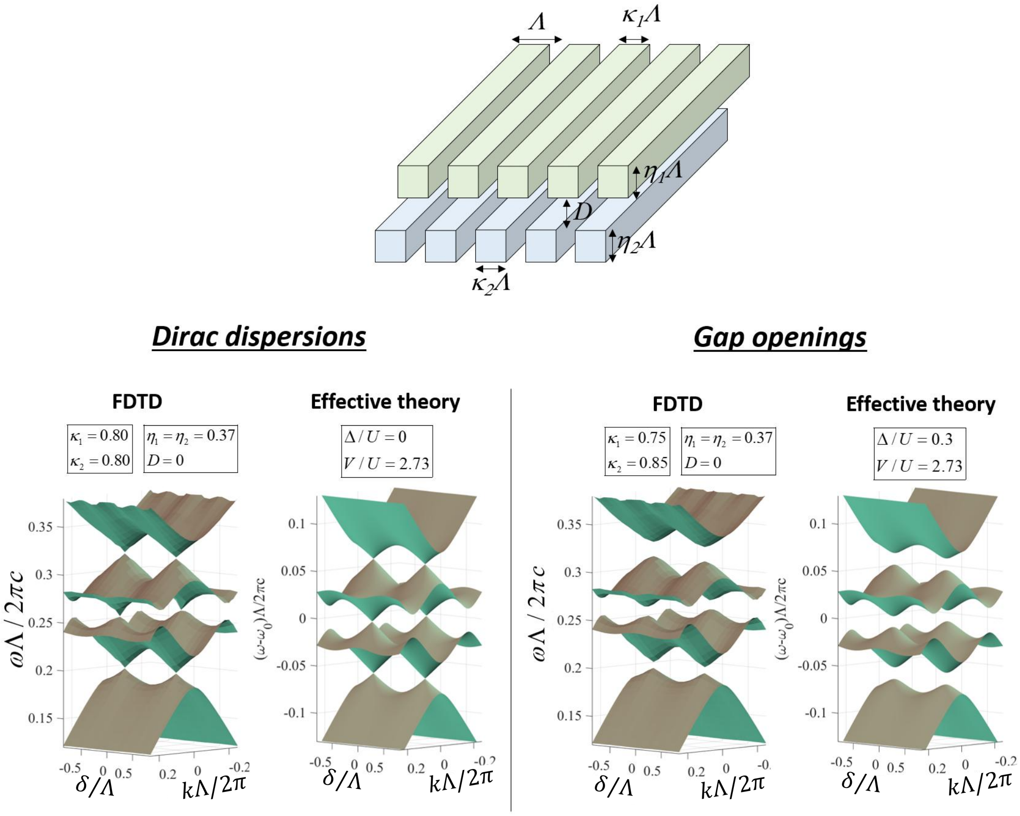

Figure S9: Comparison between the bulk band structure obtained from FDTD simulations and the one calculated by the effective theory using the retrieved parameters shown in Fig. S8. Here, the simulated band structures are only shown in the vicinity of $k = 0$ (i.e., X point of the 1D Brillouin zone) since the effective theory is only valid in this region.

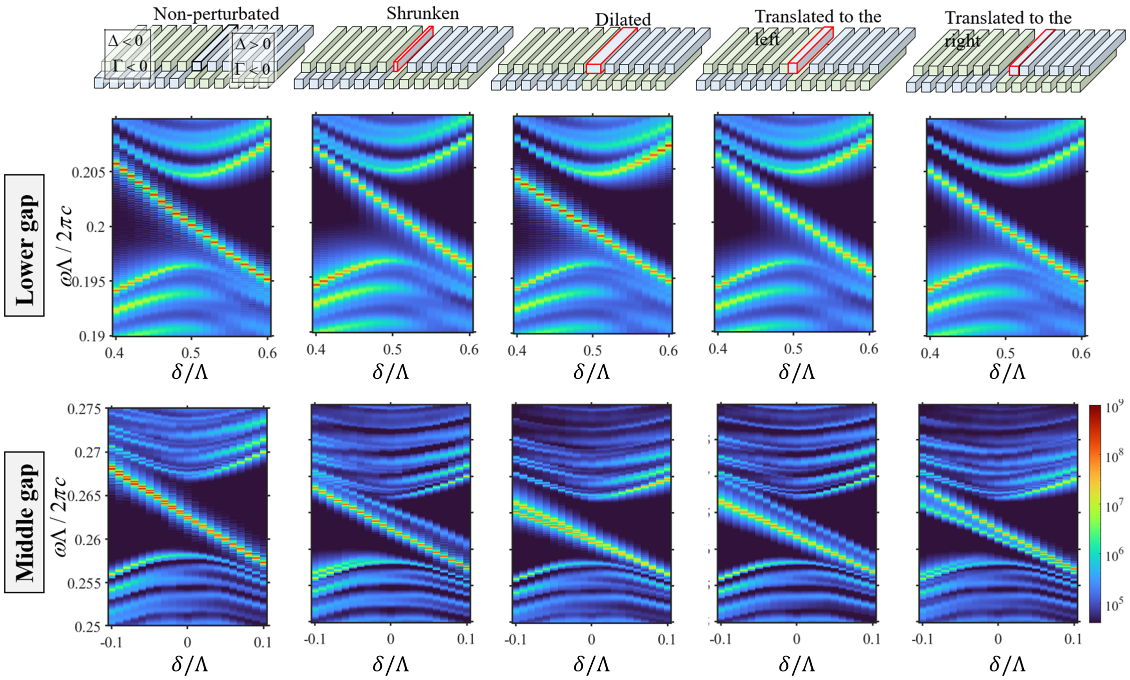

Figure S10: The robustness of the chiral edge states against defects at the interface. Here, the interface is strongly perturbed by modifying either the size or the position of the first rod in the upper-right grating. These results show that: i) the chirality is topologically protected against perturbations, and ii) the degeneracy lifting of the two edge states in the middle gap depends strongly on the interface, i.e., the boundary conditions.
